# Olfaction at depth: Cribriform plate size declines with dive depth and duration in aquatic arctoid carnivorans

**DOI:** 10.1002/ece3.6343

**Published:** 2020-06-23

**Authors:** Deborah J. Bird, Iman Hamid, Lester Fox‐Rosales, Blaire Van Valkenburgh

**Affiliations:** ^1^ Department of Ecology and Evolutionary Biology University of California Los Angeles Los Angeles CA USA

**Keywords:** aquatic adaptations, cribriform plate, diving behavioral ecology, marine mammals, olfaction, skull morphology

## Abstract

It is widely accepted that obligate aquatic mammals, specifically toothed whales, rely relatively little on olfaction. There is less agreement about the importance of smell among aquatic mammals with residual ties to land, such as pinnipeds and sea otters. Field observations of marine carnivorans stress their keen use of smell while on land or pack ice. Yet, one dimension of olfactory ecology is often overlooked: while underwater, aquatic carnivorans forage “noseblind,” diving with nares closed, removed from airborne chemical cues. For this reason, we predicted marine carnivorans would have reduced olfactory anatomy relative to closely related terrestrial carnivorans. Moreover, because species that dive deeper and longer forage farther removed from surface scent cues, we predicted further reductions in their olfactory anatomy. To test these hypotheses, we looked to the cribriform plate (CP), a perforated bone in the posterior nasal chamber of mammals that serves as the only passageway for olfactory nerves crossing from the periphery to the olfactory bulb and thus covaries in size with relative olfactory innervation. Using CT scans and digital quantification, we compared CP morphology across Arctoidea, a clade at the interface of terrestrial and aquatic ecologies. We found that aquatic carnivoran species from two lineages that independently reinvaded marine environments (Pinnipedia and Mustelidae), have significantly reduced relative CP than terrestrial species. Furthermore, within these aquatic lineages, diving depth and duration were strongly correlated with CP loss, and the most extreme divers, elephant seals, displayed the greatest reductions. These observations suggest that CP reduction in carnivorans is an adaptive response to shifting selection pressures during secondary invasion of marine environments, particularly to foraging at great depths. Because the CP is fairly well preserved in the fossil record, using methods presented here to quantify CP morphology in extinct species could further clarify evolutionary patterns of olfactory loss across aquatic mammal lineages that have independently committed to life in water.

## Graphical abstract

In a comparative study across arctoid carnivorans from terrestrial, aquatic, and semi‐aquatic ecologies, we found marked losses in olfactory skull morphology, specifically the cribriform plate, within two lineages that independently reinvaded the marine environment, Pinnipedia, and Mustelidae (sea otter). In a further investigation focusing on aquatic arctoids and the influence of highly variable diving behavioral ecology on olfactory morphology, we found that dive depth and duration are strongly correlated with cribriform plate losses. Findings suggest that reductions in olfactory anatomy in carnivorans is an evolutionary adaptive response to shifting selection pressures associated with reinvasion of marine environments, particularly to foraging “noseblind” and at great depths.

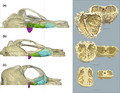

## 1 INTRODUCTION

Mammals rely on their sense of smell to varying degrees and their olfactory systems have evolved to operate in distinct ecological contexts. As lineages, foraging landscapes, and chemical stimuli change over evolutionary time, species acquire and lose olfactory capacities (Gittleman, 2013; Hayden et al., 2010; Van Valkenburgh et al., 2011). For example, it is widely accepted that obligate aquatic mammals such as odontocete cetaceans, and to a lesser extent mysticetes, have lost some degree of olfactory anatomy, genes and behaviors relative to their living terrestrial relatives and ancestors (Kishida, Thewissen, Hayakawa, Imai, & Agata, 2015; Liu et al., 2019; Oelschläger, 1992; Oelschläger & Buhl, 1985). There is less agreement on the relative role smell plays in the life of aquatic mammals with residual ties to the land, such as marine arctoid carnivorans, the pinnipeds (seals, sea lions, and walrus) and sea otter (*Enhydra lutris*). Some studies (Harrison & Kooyman, 1968; Van Valkenburgh et al., 2011) have suggested that the olfactory apparatus of pinnipeds is generally reduced relative to their terrestrial carnivoran relatives, while another study found no significant difference (Pihlström, 2008). Support for a keen sense of smell in pinnipeds and sea otters comes from field observations of scent‐driven behaviors, such as nose‐to‐nose nuzzling, genital sniffing, alarm responses to upwind biologists, and aversive reactions to con‐specific carcass odors (Lowell & Flanigan, 1980; Peterson & Bartholomew, 1967; Riedman & Estes, 1990; Ross, 1970), all of which are also observed in terrestrial carnivorans. However, there is one olfactory dimension missing from this discussion. Unlike terrestrial species, aquatic carnivorans capture prey exclusively underwater and do so “noseblind.” With nostrils closed, diving mammals are shut off from all chemical cues except those they detect at the surface (Reidenberg, 2007; Riedman & Estes, 1990). It is thought that foraging pinnipeds use surface odors, such as dimethyl sulfide (DMS), to locate areas of high marine productivity in the same way mysticete whales and sea birds do (Bouchard et al., 2019; Kowalewsky, Dambach, Mauck, & Dehnhardt, 2006; Nevitt, 1999); however, once underwater, these diving carnivorans can no longer use the landscape of chemical cues relied on by terrestrial species to locate and capture prey (Smith, 1980; Ylönen, Sundell, Tiilikainen, Eccard, & Horne, 2003). For this reason, we pose a first, general hypothesis that aquatic carnivorans rely less on olfaction than closely related terrestrial species and predict that this will be manifested in reduced olfactory anatomy.

Secondly, dietary regimes vary widely across aquatic carnivorans and include pelagic and mesopelagic cephalopods and fish, benthic invertebrates, coastal zooplankton, penguins, and pinniped pups, among others (Bowen & Siniff, 1999; Pauly, Trites, Capuli, & Christensen, 1998). Coupled with this ecological diversity, pinnipeds and sea otters have evolved a wide range of diving behaviors, both the depth at which they pursue prey and the length of time spent diving (Ponganis, 2011; Schreer & Kovacs, 1997). For example, sea otters’ dives average ca. 12 meters and last a little over a minute (Bodkin, Esslinger, & Monson, 2004; Tinker, Costa, Estes, & Wieringa, 2007), while northern elephant seals’ dives average over 500 meters and can last up to two hours (Delong & Stewart, 1991; Robinson et al., 2012). We hypothesize that this diversity in diving behavior influences olfactory capacity for two reasons. First, because deeper and more extended dives remove underwater foragers from informative surface odorant cues that might be present at a dive's initiation site (Davis, Fuiman, Williams, Horning, & Hagey, 2003; Davis, Fuiman, Williams, & Le Boeuf, 2001; Harcourt, Hindell, & Bell, 2000), we hypothesize that among aquatic carnivorans, selection for keen olfactory performance is further reduced in more extreme divers. Additionally, cranial adaptations to the challenges of diving in low light (Welsch et al., 2001) under fluctuating pressure (Kooyman, 1973) include enlarged orbits and the reduction of air‐filled skull cavities (Curtis, Lai, Wei, & Van Valkenburgh, 2015; King, 1983). These aquatic specializations likely constrain the space available for olfactory structures and tend to be more extreme in species that dive deeper (Debey & Pyenson, 2013). Consequently, we predict that within the aquatic carnivorans, reductions in olfactory anatomy will be inversely related to diving depth and duration.

**FIGURE 1:**
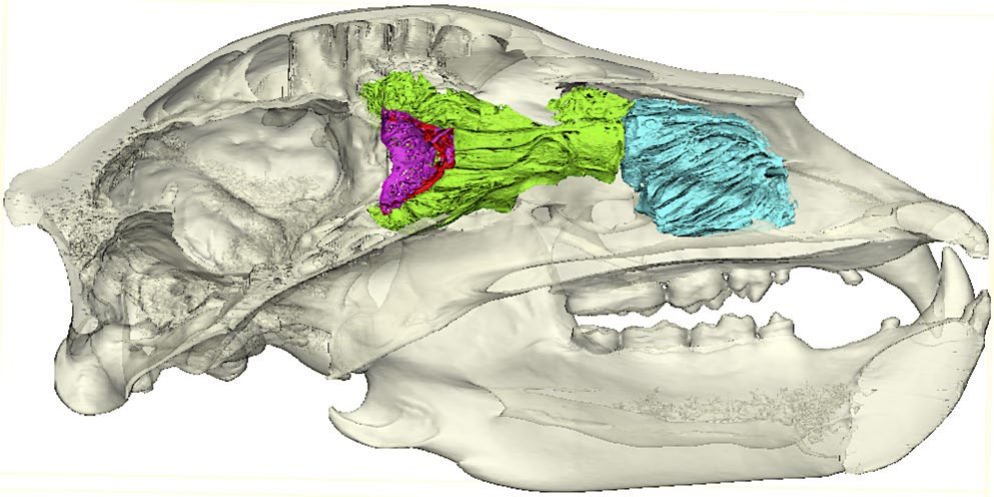
Nasal anatomy of grizzly bear (*Ursus arctos*). Left half of a sagittally sectioned skull. Pink, perforated cribriform plate bone separating nasal cavity from the brain case. Green, olfactory (ethmo‐, fronto‐ and naso‐) turbinals. Blue, respiratory (maxillo‐) turbinals

Previous work on nasal turbinals in carnivorans suggested that aquatic species had reduced olfactory turbinal surface areas relative to their terrestrial relatives (Van Valkenburgh et al., 2011). However, only five aquatic species were sampled, making this conclusion tentative, and the authors did not examine any correlations with diving behavior. To further test the impact of aquatic foraging on olfactory anatomy, we expanded the number of aquatic species sampled to 19 and examined a different metric of olfactory anatomy, the area of the cribriform plate (CP). The CP is a bone in the posterior nasal cavity of mammals that is perforated with passageways for olfactory nerve bundles crossing from the periphery to the olfactory bulb of the brain (Negus, 1958) (Figure 1).

We chose to study the CP for several reasons. First, because its size varies with the amount of peripheral olfactory innervation found in a mammal's snout (Pihlström, Fortelius, Hemilä, Forsman, & Reuter, 2005), quantifying the CP provides an opportunity to gauge and compare relative olfactory investment across aquatic and terrestrial species (Bird, Amirkhanian, Pang, & Van Valkenburgh, 2014). Second, earlier work found that, across all superorders of mammals, relative CP size is closely correlated with the number of olfactory receptor genes in an animal's genome, thereby establishing CP morphology as an informative metric of relative reliance on the sense of smell (Bird et al., 2018). Third, CP area is tightly correlated with the surface area of the ethmoturbinals, the bony plates that bear olfactory epithelium (Bird et al., 2014). Finally, CP area can be quantified in some fossil skulls (Bird et al., 2018), and so will enable future studies into the evolution of olfactory anatomy in extinct mammal lineages that have transitioned from land to water. Here, we perform the first extensive comparative and quantitative study of the CP morphology of arctoid carnivorans, a clade that has seen multiple independent invasions into the marine habitat and includes species at the intersection of terrestrial and aquatic life.

**FIGURE 2:**
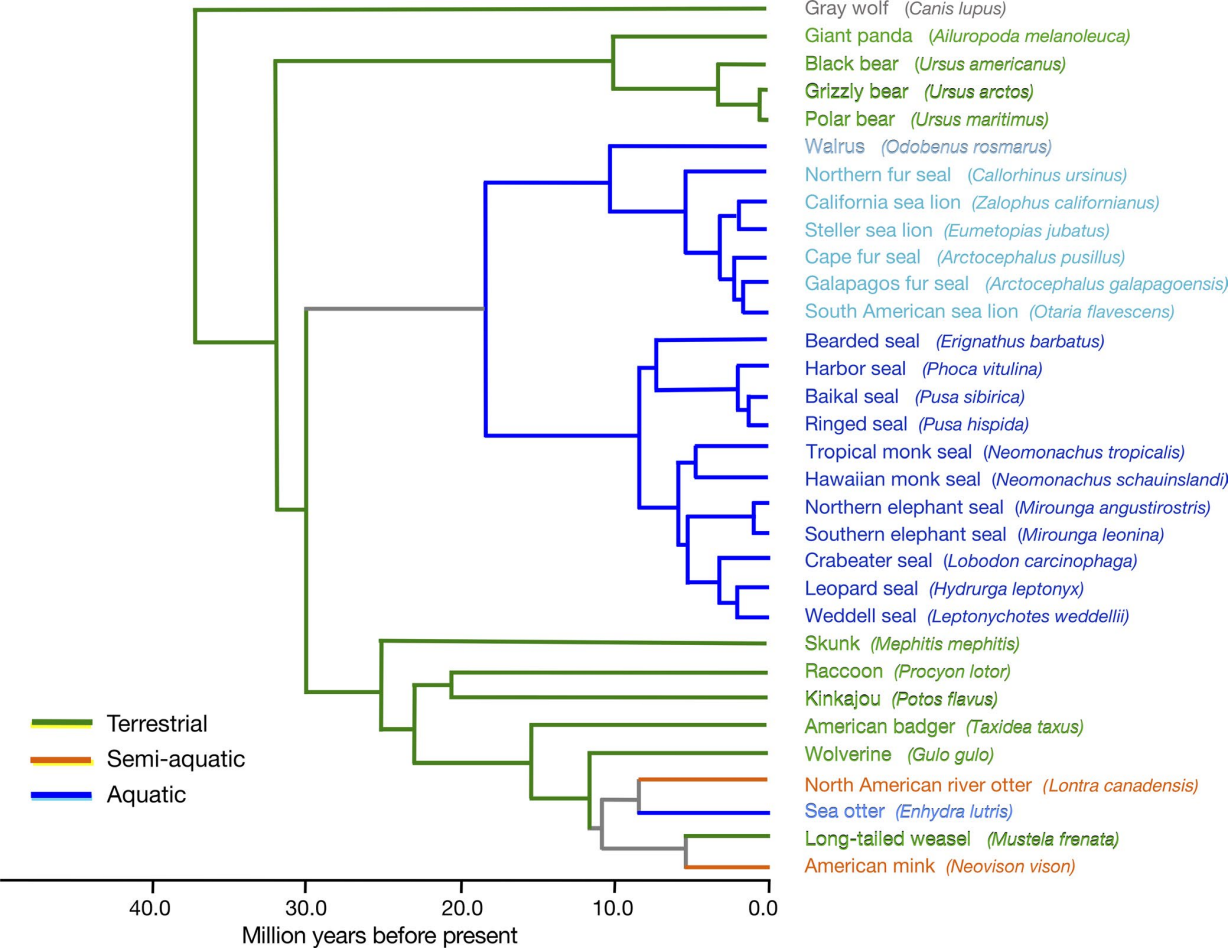
Time‐calibrated phylogeny for arctoid carnivorans. All taxa except the gray wolf were sampled for this study. Topology and divergence estimates are taken from Upham et al. (2019)

Our sample group, the arctoid carnivorans, is an ecologically rich clade that includes ursids (bears), mustelids (e.g., weasels, otters, and badgers), procyonids (e.g., raccoons and kinkajous), mephitids (skunks), and pinnipeds, among others (Figure 2) (Upham, Esselstyn, & Jetz, 2019). Within the arctoids, there were multiple independent, secondary entries into aquatic habitats (Berta, Sumich, & Kovacs, 2015), resulting in a diversity of closely related species from disparate ecologies (aquatic, semi‐aquatic, and terrestrial) along a spectrum of olfactory demands. According to the recent comprehensive mammalian phylogenetic analysis (Upham et al., 2019), pinnipeds diverged from the lineage leading to Musteloidea ca. 24–33 ma., and otters diverged from terrestrial mustelids more recently, ca. 8.5–12 million years ago. Studying Carnivora is advantageous, as the group has a fairly well‐resolved phylogeny, allowing the application of comparative methods that account for phylogenetic relatedness in our study of ecological influences on olfaction.

## 2 MATERIALS AND METHODS

Using high‐resolution CT scans and 3D imaging software and methods developed in previous studies (Bird et al., 2014, 2018), we measured the surface area of the perforated region of the CP as well as the cumulative cross‐sectional area of the CP foramina as proxies for relative olfactory innervation found in individual arctoid species.

### 2.1 Specimen collection

We sampled 65 skulls from 31 species representing eight families of arctoid carnivorans (Figure 2) (Upham et al., 2019). Specimens and their source museums are listed in Table A1 in Appendix. All species are extant with the exception of the tropical monk seal (*Neomonachus tropicalis*). Body sizes span several orders of magnitude from <1 kg (long‐tailed weasel, *Mustela frenata*) to at least 1,275 kg (male southern elephant seal, *Mirounga leonina*) (Irvine, Hindell, Van Hoff, & Den, 2000). Where possible, we sampled two wild‐caught adult specimens, one male and one female, for each species.

### 2.2 Morphological data

**FIGURE 3:**
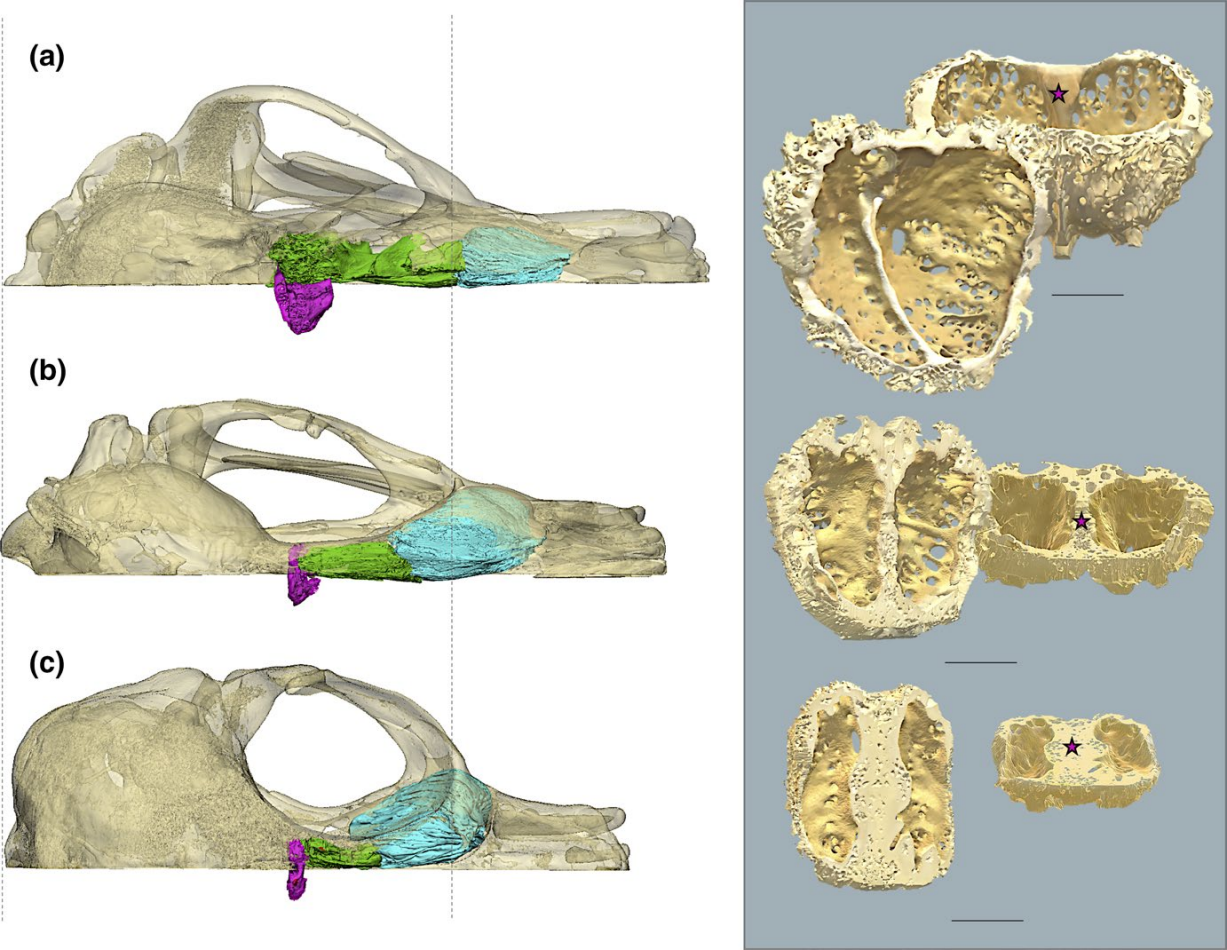
Cribriform plate morphology in terrestrial and aquatic carnivorans. (a) grizzly bear (*Ursus arctos*); (b) leopard seal (*Hydrurga leptonyx*); (c) northern elephant seal (*Mirounga angustirostris*). Left panel, dorsal view of left half of sagittally sectioned skull. Pink, entire cribriform plate (CP). Green, left olfactory turbinals. Blue, left respiratory turbinals. Dashed lines indicate landmarks for measurement of occipital condyle to orbit distance. Right panel: CP of each species enlarged. Left, posterior oblique view. Right, ventral oblique view: note large, densely perforated roof concavity in grizzly CP. Star, crista galli. Scale bar, 10 mm

**FIGURE A1:**
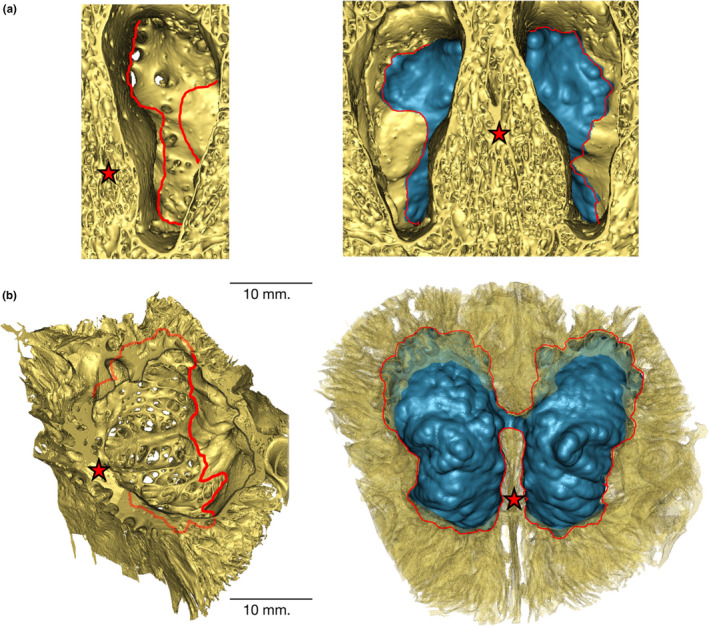
Quantifying the cribriform plate surface area. 3D digital rendering of (a), Northern elephant seal (*Mirounga angustirostris*) and (b), grizzly bear (*Ursus arctos*) cribriform plates (CP), constructed in Mimics and viewed here in posterior aspect from the brain case. Left: right half of CP viewed in oblique angle; the region perforated by olfactory foramina is delineated (red). Right: full CP, posterior view; olfactory foramina are digitally filled and the generalized surface of the perforated region (blue) is calculated in 3‐matic. The full grizzly bear CP (b, right) is rendered transparent in order to better view its deep concavity. Star: crista galli (CP midline)

Thirty‐five of the 65 skulls were scanned at the University of Texas High Resolution CT Scanning Facility (http://www.ctlab.geo.utexas.edu). The remaining 30 skulls were scanned on Phoenix nanotom s™ and Nikon Metrology XT H 225 ST machines at the Molecular Imaging Center of the Keck School of Medicine of USC in Los Angeles, on Phoenix v|tomex™ machines at General Electric's Inspections Technologies Facility in San Carlos, California, or on a Siemens Definition AS64™ scanner at Ronald Reagan Medical Center at UCLA. In order to maximize resolution, the field of view was restricted to the CP area of the skull in most cases, although a number of skulls were scanned in their entirety. Voxel size ranged from 0.044 to 0.5 mm. All scans are available upon request from either Digimorph (http://www.digimorph.org) or MorphoSource (http://www.morphosource.org/). Scans were imported into the 3D imaging software Mimics (v. 15.0‐21.0, Materialise, Leuven, Belgium), segmented into two dimensional masks, and reconstructed as volumetric renderings. Edited 3D models of the CP constructed for each specimen could be rotated and magnified for closer inspection and quantification. When needed, multiple regions of interest in the skull were segmented and rendered as separate 3D models to better visualize the CP in the context of its surrounding nasal anatomy (Figures 1 and 3). The first metric, CP surface area, includes only the section of the CP bone perforated by foramina that surround the olfactory nerves. We quantified CP surface area by generating a continuous surface in the imaging program 3‐matic (v. 7.01‐13.0, Materialise) with a wrapping function that fills all foramina in the CP model, then digitally cutting the surface at the perimeter of the perforated region and calculating its area in 3‐matic (Bird et al., 2018) (Figure A1). To quantify the cumulative cross‐sectional area of individual CP foramina, our second metric, we applied splines, or rings of coordinate points, to the perimeters of the CP foramina in Mimics. We imported the resulting splines into modeling software Rhinoceros‐4 (McNeel and Associates), where surface areas for all foramina were calculated and tallied. While total foramina area may be the most direct estimate of the cross‐sectional area of an animal's olfactory innervation, it cannot be resolved from low resolution scans, damaged skulls, or fossils. Therefore, because foramina area is closely correlated with CP surface area (*r* ^2^ = .92; pgls‐*r* ^2^ = .9, *p* < .001, Figure A3), we used the latter to maximize sample size.

As a body size proxy, we used the skull metric, occiput‐to‐orbit length (OOL), defined as the distance between the posterior extent of the occipital condyles and the anterior most extent of the orbit (Figure 3). The correlation between OOL and body mass is similar to that between full skull length and body mass (Van Valkenburgh, 1990), and OOL offers advantages over skull length. First, OOL excludes the confounding influence of snout length, a feature that varies widely among arctoids independent of body size. Indeed, in our sample, relative snout lengths are shorter in aquatic species than terrestrials (*p = *.036, Table A1 and A3). OOL also allows the inclusion of skulls with broken premaxillae and is better suited to analyze incomplete fossil skulls in the future. Using the skull metric OOL instead of body mass reduced the excessive influence of large fat stores in pinnipeds on body size estimates. For all specimens, in our sample, the skull metric OOL was measured from 3D skull reconstructions using Mimics or from skulls directly using digital calipers.

### 2.3 Habitat groupings

We grouped the arctoids into three ecological categories, terrestrial, aquatic, and semi‐aquatic. We defined terrestrial species as those that live and forage exclusively on land. These include ten species of ursids, procyonids, mephitids, and mustelids (Figure 2, Table A1). Species in the aquatic group (*n* = 19) forage exclusively underwater but also spend some time hauled out on land or pack ice and include eighteen pinniped species and the mustelid sea otter (*Enhydra lutris*). Semi‐aquatic species forage both underwater and on land and include two mustelid species. Although the polar bear (*Ursus maritimus*) is often referred to as a semi‐aquatic marine mammal and sometimes swims to stalk its prey (Berta et al., 2015), we chose to classify it as terrestrial, as it does not seek and capture its prey underwater (Stirling, 1974).

### 2.4 Diving data

Four diving behavior variables are included in this study, maximum dive depth, mean dive depth, maximum dive duration, and mean dive duration. All dive data were compiled from published behavioral field studies (Table A2). If means were not directly reported in source literature, we derived these from supplemental raw dive data, data shared in personal communications, or in two cases by visually measuring from histogram distributions. We included as many studies as possible in calculating our means, weighting the contribution of each study by the number of animals recorded. Other potentially informative variables describing potential diving capacity or overall degree of aquatic specialization, such as magnitude and distribution of oxygen stores, at‐sea durations, migration distances, haul‐out durations, exist for some but not all sample species, and so could not be used for this study.

### 2.5 Statistical analysis

Species means of all morphological and ecological variables were used for data analysis. To view scaling relationships between CP and body size and to derive values for size‐adjusted relative CP size, we plotted log_10_ absolute CP surface area against log_10_ OOL using phylogenetic least squares regression (PGLS). Resulting residuals were used as relative CP size (RelCP) in all subsequent analyses. To test the influence of habitat on RelCP values, we performed pair‐wise ANOVA and Tukey HSD post hoc tests. All regression plots include regression lines from PGLS as well as general least squares regression (GLS). All analyses were performed in R (Team RC, 2015). For PGLS, we used Caper Package (Orme et al., 2013) and a time‐calibrated mammal tree pruned to include only the species in our study (Upham et al., 2019).

## 3 RESULTS

### 3.1 Cribriform plate area and body size

**FIGURE 4:**
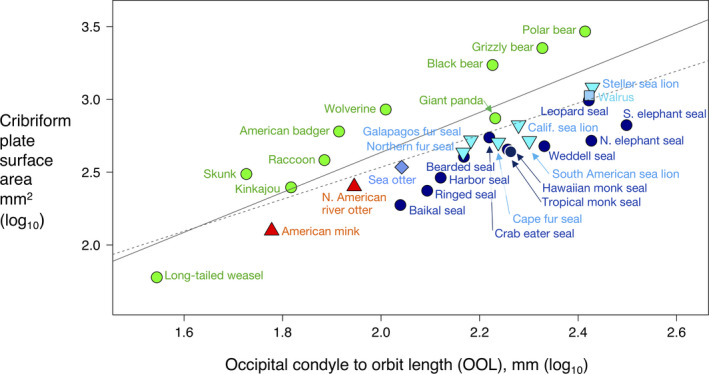
Log–log plot of CP surface area versus Occiput‐orbit length (OOL) for three ecological groupings. Green circles, terrestrial species; red triangles, semi‐aquatics; dark blue circles, Phocidae; turquoise inverted triangles, Otariidae; blue diamond, Mustelidae (sea otter, *Enhydra lutris*); light blue square, Odobenidae (walrus, *Odobenus rosmarus*); Solid line, best fit from phylogenetic generalized least squares (PGLS) regression; dotted line, best fit from generalized least squares regression (GLS)

Among all 31 species, absolute CP surface area is coupled to body size, as described here by the skull metric, occipital condyle to orbit length (OOL) (pgls‐*r* ^2^ = .7, *p* < .001), and scales with negative allometry (*y* = 1.37*x* − 0.1097), (Figure 4, Table A3). Thus, large species have proportionally smaller CP for their body size. There is considerable scatter about the line with terrestrial species tending to fall above the line and aquatics below the line. Among the aquatic species alone the relationship between CP surface area and OOL is similar (pgls‐*r* ^2^ = .69, *p* < .001, *n* = 18) and among terrestrials alone it is stronger (pgls‐*r* ^2^ = .84, *p* < .001, *n* = 10).

### 3.2 Cribriform plate in terrestrial, aquatic, and semi‐aquatic species

To test the hypothesis that aquatic and semi‐aquatic species have reduced olfactory morphology relative to terrestrial species, we performed a one‐way ANOVA and Tukey HSD post hoc tests on mean relative CP size (RelCP) values from all three habitat groups. Aquatic species have significantly smaller mean RelCP than terrestrial species (*p < *.001). Mean RelCP of semi‐aquatic species is smaller than that of terrestrials (*p = *.014) and does not differ significantly from that of aquatics (*p = *1) (Table A3).

**FIGURE A2:**
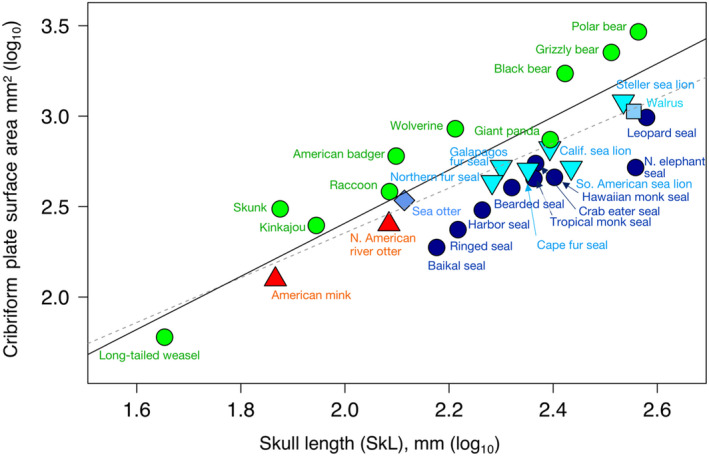
Log‐Log plot of CP surface area vs. full skull length (occipital condyle to prosthion)(SkL) for three ecological groupings. Green circles, terrestrial species; red triangles, semi‐aquatics; blue, aquatics; dark blue circles, Phocidae; turquoise inverted triangles, Otariidae; blue diamond, Mustelidae (sea otter, *Enhydra lutris*); light blue square, Odobenidae (walrus, *Odobenus rosmarus*); Solid line, best fit from phylogenetic generalized least squares (PGLS) regression; dotted line, best fit from generalized least squares regression (GLS)

**FIGURE A3:**
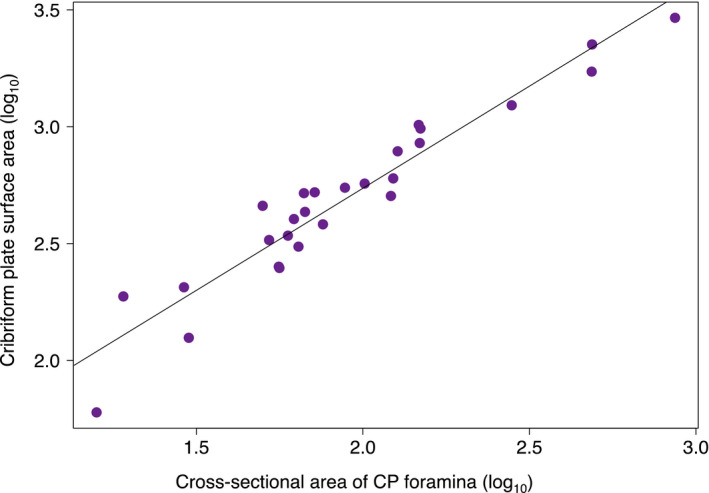
Log‐log regression plot of CP surface area vs. cumulative cross‐sectional area of CP foramina (mm^2^) for 26 arctoid species, (*r* ^2^ = .92, pgls‐*r* ^2^ = .9, *p <* .001).

Similarly, when running a habitat analysis on CP surface area that is size‐adjusted to full skull length (SkL) instead of OOL, comparable differences between groupings emerge (Appendix A1, Figure A2). As per Tukey HSD post hoc tests, again aquatics have significantly larger mean RelCP than terrestrials (*p < *.001), and there is no significant difference between semi‐aquatics and aquatics (*p = *.36). The difference in mean RelCP (size‐corrected to full skull length) between semi‐aquatic species and terrestrials is less pronounced but significant (*p = *.042).

To consider whether the losses in olfactory anatomy in the aquatic mustelids occurred independently from those in the lineage leading to Pinnipedia, we analyzed RelCP in aquatics and terrestrials within the clade Musteloidea (mustelids, procyonids, and mephitid; *n = *9) and within the family Mustelidae (*n = *6) separate from Pinnipedia and Ursidae. A phylogenetically corrected ANOVA shows that the mean RelCP of the aquatic sea otter and semi‐aquatic river otter and mink together are significantly smaller than the mean RelCP of terrestrial musteloids (*p = *.007) and terrestrial mustelids (*p = *.014). It is interesting that among the three terrestrial mustelid species, the long‐tailed weasel differs from the much larger badger and wolverine by having a reduced RelCP similar to that of the three more aquatic mustelids. This suggests that a reduced RelCP might be characteristic of smaller mustelids in general. Without a larger sample size of small mustelids, the diminutive RelCP of the long‐tailed weasel is difficult to interpret.

### 3.3 RelCP and diving ecology of aquatic carnivorans: dive depth and duration

**FIGURE 5:**
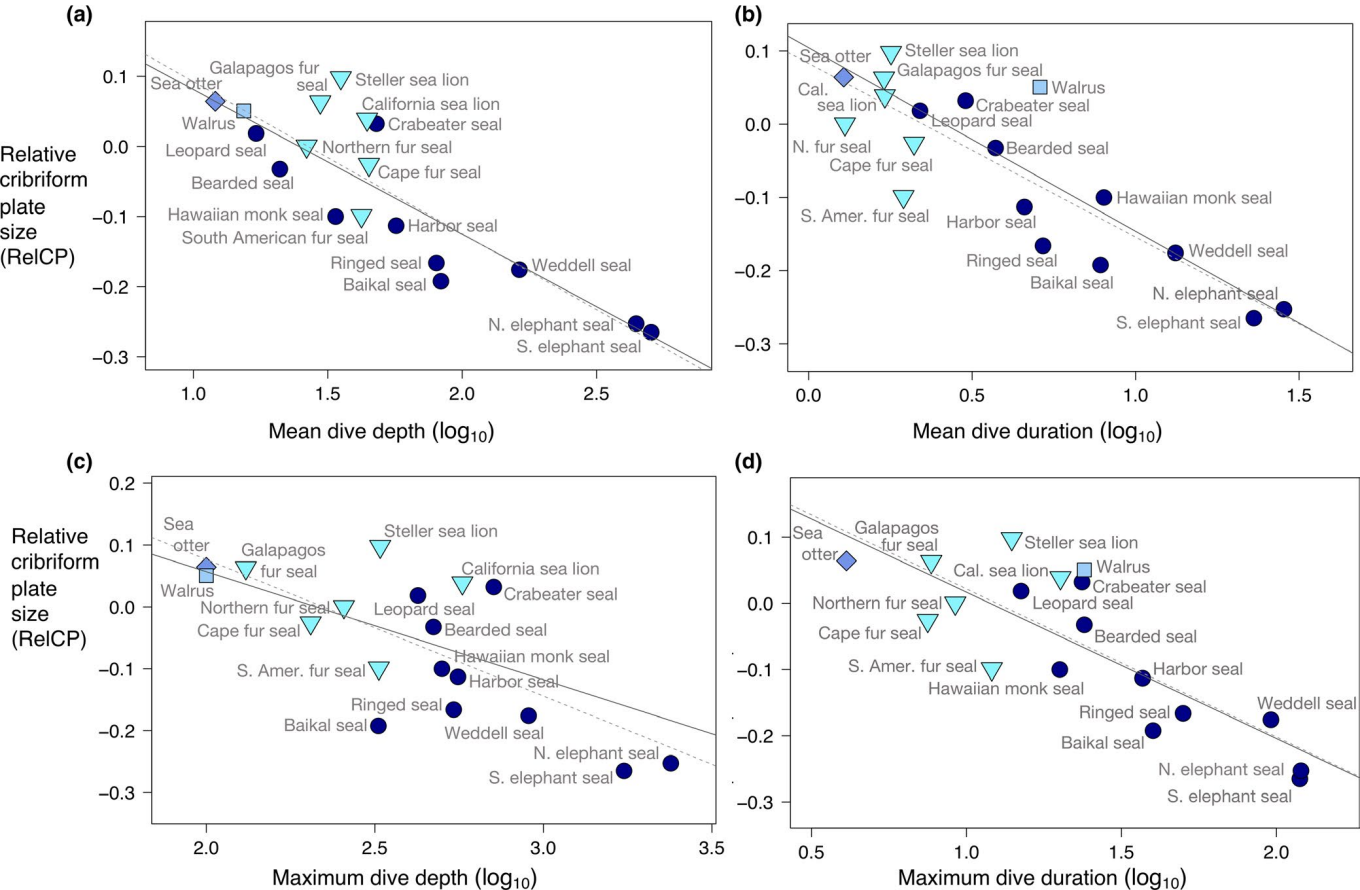
Significant correlation between relative cribriform plate size and three dive variables in the four families of aquatic carnivorans. (*a*) Relative CP size (RelCP) versus mean dive depth (*r* ^2^ = .75, *p < *.001, pgls‐*r* ^2^ = .65, *p* < .001). (b) RelCP versus mean dive duration (*r* ^2^ = .76, *p < *.001, pgls‐*r* ^2^ = .61, *p* < .001). (c) RelCP versus. maximum dive depth (*r* ^2^ = .55, *p < *.001, pgls‐*r* ^2^ = .22, *p* = .51). (d) RelCP versus. maximum dive duration (*r* ^2^ = 0.66, *p < *.001, pgls‐*r* ^2^ = .48, *p* < .001). Dark blue circles, Phocidae; turquoise inverted triangles, Otariidae; light blue square, Odobenidae (walrus); blue diamond, Mustelidae (sea otter). Solid line, best fit from PGLS regression; dotted line, best fit from GLS regression

**FIGURE A4:**
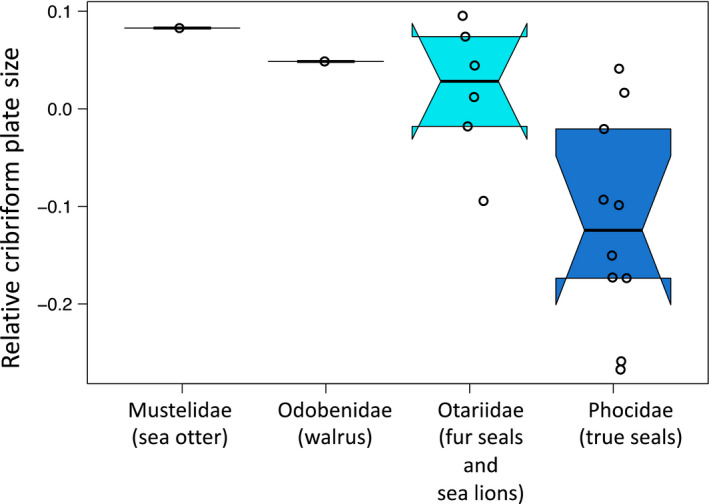
The influence of phylogeny on relative cribriform plate size among aquatic carnivorans. While there is overlap across all four families, the phocids tend to have, on average, smaller cribriform plates than the otariids, as well as the odobenid and mustelid. See Table S3 Summary Statistics.

To investigate possible interactions between diving behavior and olfactory morphology, we tested for correlations between RelCP and each of four diving parameters, mean dive duration, maximum dive duration, mean dive depth, and maximum dive depth within the 18 aquatic species for which we had published dive data (17 pinnipeds and the sea otter). We found strong inverse relationships between RelCP and three of the variables, mean dive depth (*r* ^2^ = .75, *p < *.001, pgls‐*r* ^2^ = .65, *p* < .001), mean dive duration (*r* ^2^ = .76, *p < *.001, pgls‐*r* ^2^ = .61, *p* < .001), and maximum duration (*r* ^2^ = .66, *p < *.001, pgls‐*r* ^2^ = .48, *p* < .001), respectively (Figure 5a,b,d),. This relationship is largely driven by the phocids; in all three cases, accounting for phylogeny weakens the coefficients of determination because the otariids tend not to follow the main trend and phylogeny exerts a strong influence on pinniped CP morphology independent of diving behavior. Phocids have on average smaller RelCP than either otariids alone (*p = *.015) and otariids and the odobenid walrus together (*p = *.019) (Figure A4, Table A3). In the case of the fourth parameter, maximum dive depth, what appears to be a strong negative relationship with RelCP (*r* ^2^ = .55, *p < *.001), is barely significant after accounting for phylogeny (pgls‐*r* ^2^ = .22, *p = *.051) (Figure 5c, Table A3).

**FIGURE 6:**
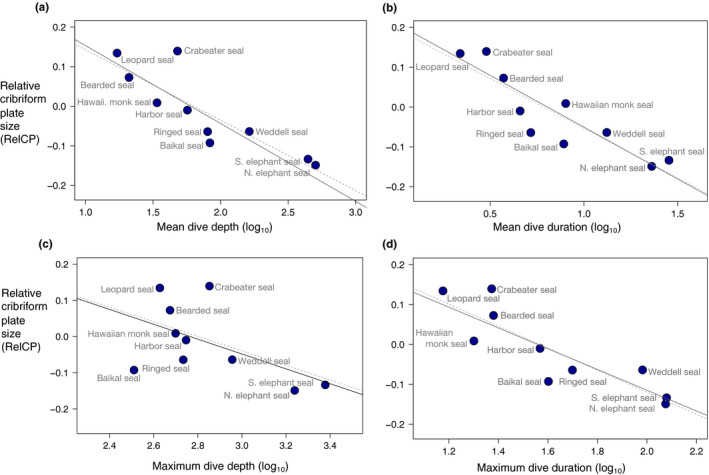
Relationship between RelCP and dive behavior among the phocids. Regression plots of relative CP size versus (a) mean dive depth (*r* ^2^ = .75, *p < *.001, pgls‐*r* ^2^ = .82, *p* < .001); (b) mean dive duration (*r* ^2^ = .79, *p < *.001, pgls‐*r* ^2^ = .88, *p* < .001); (c) maximum dive depth (ns: *r* ^2^ = .3, *p = *.1, pgls‐*r* ^2^ = .22, *p* = .17); (d) maximum dive duration (*r* ^2^ = .76, *p < *.001, pgls‐*r* ^2^ = .78, *p* < .001). Solid line, best fit from PGLS regression; dotted line, best fit from GLS regression

The true seals, phocids, display a far wider range of mean dive depths (~17–505 m) and mean dive duration (~3–28 min) than their sister clade of otariids and odobenids (15.4–44 m, 1.7–5.1 min, respectively) (Figures 5 and 6a,b, Table A2), suggesting more extensive ecological diversity among the true seals, and so we examined them separately. We calculated RelCP values for phocids alone using residuals from the PGLS regression of CP surface area against OOL among the ten phocid species. Within the phocids, there is a strong negative correlation between RelCP and three diving metrics: mean dive depth (*r* ^2^ = .75, *p < *.001, pgls‐*r* ^2^ = .82, *p* < .001), mean dive duration (*r* ^2^ = .79, *p < *.001, pgls‐*r* ^2^ = .88, *p* < .001), and maximum dive duration (*r* ^2^ = .76, *p < *.001, pgls‐*r* ^2^ = .78, *p* < .001) but no significant relationship with maximum dive depth (Figure 6a–d, Table A3).

## 4 DISCUSSION

Results from our study point to reduced reliance on olfaction as a secondary adaptation to marine habitats, and in particular to foraging at depth. Among aquatic arctoid carnivorans, we found a pronounced loss of olfactory anatomy, specifically a reduction in relative cribriform plate size (RelCP), that mirrors established reductions in olfactory turbinal surface area (Van Valkenburgh et al., 2011). It is not surprising that both CP and olfactory turbinal surface areas are reduced in aquatic species, given their common developmental origin (Rowe, Eiting, Macrini, & Ketcham, 2005), and the fact that they are strongly correlated in size across all carnivorans (Bird et al., 2014). Our results accord with initial genomic studies reporting losses in the number of functional olfactory receptor genes in five aquatic arctoid species (two otter and three pinniped species) relative to terrestrial relatives (Beichman et al., 2019; Hughes, Gang, Murphy, Higgins, & Teeling, 2013; Liu et al., 2019). Our data show that pinnipeds and the sea otter, representing two lineages within Carnivora that independently reinvaded the marine environment, have likely undergone convergent reductions in relative cribriform plate size (RelCP) compared with closely related terrestrial species. Moreover, our findings go beyond previous work in revealing that, among aquatic carnivorans, species that dive deeper and for longer periods of time tend to have an even greater reduction in CP size.

Our finding of a smaller RelCP among aquatic arctoid carnivorans contradicts earlier work that concluded that CP size did not differ between pinnipeds and terrestrial carnivorans (Pihlström et al., 2005, 2008). There are several likely reasons for differences in our findings. First, Pihlström et al. (2005) used linear measurements to calculate CP surface area, whereas we relied on digital quantification, a method that better captures the highly irregular shape of the CP (Bird et al., 2014). Second, their body size proxy, skull area, does not exclude snout length, which can lead to the underestimation of body size in the typically short‐snouted pinnipeds and sea otter and a consequent inflation of size‐adjusted CP size in aquatic species. Third, our sample represents a wider sampling of pinnipeds including species with relatively small CP, such as the northern and southern elephant seals (*Mirounga angustirostris, M. leonina), *the Weddell seal (*Leptonychotes weddellii*), Baikal seal (*Pusa sibirica*) and ringed seal (*Pusa hispida*). 

Within aquatic carnivorans, we found marked variation in olfactory morphology that corresponds closely with diving behavior. Although there is no clearly significant association between maximum dive depth and RelCP that persists after phylogenetic accounting, there are strong inverse correlations between RelCP and the following three dive variables: mean dive depth, mean dive duration, and maximum dive duration. These relationships are even more pronounced when considering the phocids, or true seals, alone. The absence of a significant relationship between maximum dive depth and RelCP was surprising given that maximum dive depth values exhibit the widest range of the four diving parameters and because the smallest RelCP values by far belong to the most extreme divers, the northern and southern elephant seals, which have been recorded diving to 1,735 and 2,388 m, respectively (Costa et al., 2010; Costa, Robinson, et al., 2010; Robinson et al., 2012). However, close review of the published literature reveals that recorded maximum depths are often not representative of species’ overall diving patterns. For example, the California sea lion has been recorded at a depth of 575 m, and yet this otariid is generally considered a moderately shallow diver (Berta et al., 2015; Costa, Kuhn, & Weise, 2007).

Why might selection favor smaller RelCP, reduced olfactory anatomy, in aquatic carnivorans in general and species performing deeper, sustained dives in particular? We present alternative evolutionary explanations. First, although odor cues play an important role in social interactions and predator defense among aquatic carnivorans above water (Lowell & Flanigan, 1980; Peterson & Bartholomew, 1967; Riedman & Estes, 1990; Ross, 1970), below water, where pinnipeds and sea otters typically forage, odor cues are no longer detectable. Although another semi‐aquatic mammal, the water shrew, is known to exhale and inhale bubbles to access scent cues from food surfaces underwater (Catania, Hare, & Campbell, 2008), this behavior has been hypothesized but not tested in the carnivoran river otter (Marriott et al., 2013). Unlike terrestrial carnivorans, which follow deposited and airborne prey scents to locate food sources (Smith, 1980; Ylönen et al., 2003), aquatic carnivorans forage with shut nostrils and locate prey without scent cues, except those detected above water upon surfacing. Utilizing scent cues at or above the water surface is a probable tool of foraging at sea, given that harbor seals (*Phoca vitulina*) have shown keen sensitivity to dimethyl sulfide (DMS), a volatile phytoplankton odorant and indicator of local marine productivity that is utilized by sea birds as well (Kowalewsky et al., 2006; Nevitt, Reid, & Trathan, 2004). However, deeper and more extended dives increase the diver's distance from these informative odor cues at the surface (Davis et al., 2001, 2003). Consequently, over time, as selective pressure for detecting prey via odorant cues was relaxed, olfactory systems among carnivorans adapting to life in water likely decreased in size. Reduced olfactory structures were further favored because olfaction is a costly sensory system made up of millions of continually self‐replacing olfactory sensory neurons (Graziadei & Graziadei, 1985).

A second and related explanation for the reduction of olfactory anatomy in diving aquatic mammals focuses on the evolution of keen alternative sensory specializations adapted to underwater foraging. For example, pinnipeds and the sea otter possess a tactile acuity exhibited in dense arrays of highly innervated vibrissae, the most prominent of which are the mystacial (mustache) whiskers (Berta et al., 2015). Such compact arrangements of whiskers in the phocid bearded seal (*Erignathus barbatus*) and the walrus (*Odobenus rosmarus*) are thought to assist in locating mollusks in the shallow benthic substrate (Marshall, Amin, Kovacs, & Lydersen, 2006). Experiments using blindfolded animals revealed that harbor seals (*Phoca vitulina*) use their vibrissae to track the hydrodynamic trails of swimming fish (Dehnhardt, Mauck, & Bleckmann, 1998). In addition to enhanced vibrissae, aquatic carnivorans rely on a visual system adapted for hunting in dark waters. Visual specializations may include spherical lenses (Berta et al., 2015), wide pupil size range (Levenson & Schusterman, 1999) a tapetum lucidum (Kröger & Katzir, 2008) as well as proportionally large eye orbits (Debey & Pyenson, 2013). Considering these enhanced sensory specializations, it seems likely that the reduction of olfactory anatomy in pinnipeds and the sea otter over time stems, in part, from relaxed selective pressures on olfactory acuity as aquatic species come to rely more heavily on alternative sensory systems for underwater foraging.

Why does selection favor a greater reduction in CP size in aquatic carnivorans performing longer and deeper average dives? There are a number of alternative hypotheses, all of which require further testing. First, it is possible that shallow, short dive patterns reflect a closer tie to the land/pack ice, while deeper and longer dive patterns represent a more pronounced separation from a terrestrial ecology. Longer separation from land, that is, a more aquatic life, likely results in increased disconnection from airborne and deposited odor cues that terrestrial animals rely on for food, predator protection, social communication, and reproduction. To further test whether deeper, more sustained diving reflects a more fully aquatic lifestyle and an increasingly remote relationship to the land, all four diving variables might be viewed in relationship to other ecological proxies for relative proximity to land/sea ice. These factors could include foraging trip duration (Kooyman & Gentry, 1986), long‐distance migration patterns (Costa, Huckstadt, et al., 2010; Costa, Robinson, et al., 2010), pupping season duration (Stirling, 1983), haul‐out patterns (Cunningham et al., 2009) and overall at‐sea duration (Costa, Huckstadt, et al., 2010; Costa, Robinson, et al., 2010), among others. 

An alternative, or complementary, interpretation for the negative relationship between diving depth/duration and RelCP suggests that volatile odor cues at the water's surface emitted by underwater prey play a role in prey detection for marine carnivorans, and that reliance on these surface odorants selects for retention of a larger olfactory system in shallow as opposed to deep divers. One such volatile, mentioned above, is dimethylsulfide (DMS), an odorant emitted by phytoplankton, particularly when grazed upon by krill and other zooplankton (Dacey & Wakeham, 1986). Concentrations of DMS at the sea–air interface are variable, and “hotspots” indicate underlying primary production, including the presence of krill and krill‐feeding animals, such as fish or penguins (Barnard, Andreae, Watkins, Bingemer, & Georgii, 1982). The leopard seal (*Hydrurga leptonyx*), a shallow and short diver that feeds primarily on krill, penguins, and crabeater seal pups (Pauly et al., 1998), likely navigates a rich landscape of scent cues at the water's surface as well as on the ice sheet. By contrast, deep divers appear to have less access to surface cues while foraging. For example, the northern elephant seal dives in a staggered stair–step pattern, reaching its prey of pelagic squid and mesopelagic fish (Pauly et al., 1998) at depths between ~300 and 1,500 m, displaced horizontally, sometimes by hundreds of meters, from the dive initiation location (Davis et al., 2001). It is notable then, that the shallow‐diving leopard seal and the deep‐diving elephant seal, close relatives among the pinnipeds, have the most disparate RelCP among all phocids (Figure 6a). To strengthen the argument that surface odorant cues influence foraging behavior in some aquatic carnivorans, future behavioral experiments, such as those used to test responsiveness of seabirds and whales to variable concentrations of DMS (Bouchard et al., 2019; Nevitt, Veit, & Kareiva, 1995), may be performed on pinnipeds and sea otters.

**FIGURE A5:**
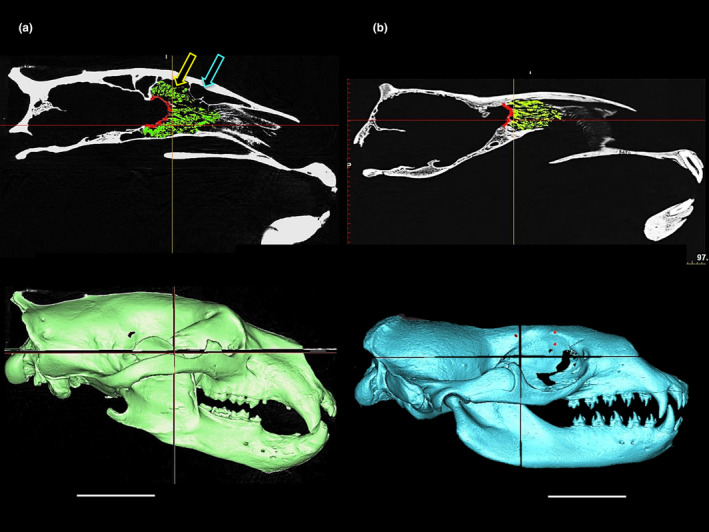
Dorsal distribution of olfactory turbinals in terrestrial and aquatic carnivorans. (a) Terrestrial carnivoran, polar bear (*Ursus maritimus*) skull in lateral aspect. Top, sagittal section at skull midline from CT scan. Green, olfactory ethmoturbinals. Red, cribriform plate. Blue open arrow, frontal sinus with no turbinals. Yellow open arrow, ethmoturbinals that have invaded the frontal sinus. Bottom, 3D digital model (b), Aquatic carnivoran, leopard seal (*Hydrurga leptonyx*) skull. Top, sagittal section of skull from CT scan. Where turbinals extend dorsally into the frontal sinus in the polar bear, there is no frontal sinus in the leopard seal skull and no dorsal projection of the olfactory turbinals (green). Scale bars, 100 mm.

**FIGURE A6:**
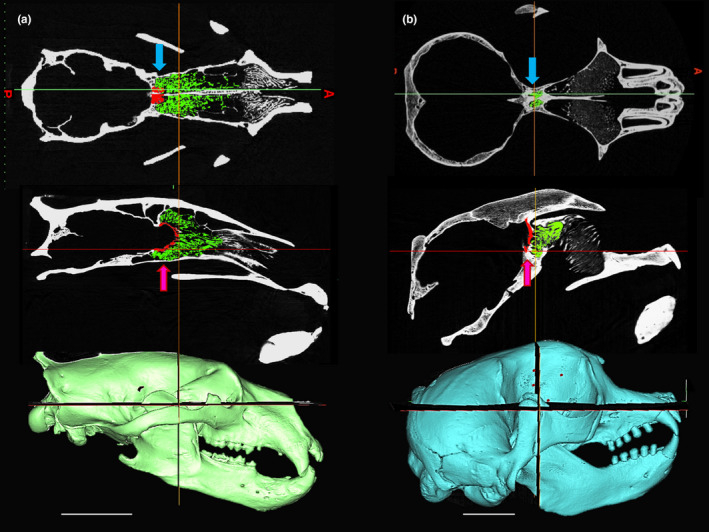
Lateral distribution of olfactory turbinals in terrestrial and aquatic carnivorans. (a) The terrestrial polar bear (*Ursus maritimus*) skull. Top, dorsal view of CT scan; middle, sagittal section at skull midline. Green, olfactory ethmoturbinals. Red, cribriform plate. Bottom, 3D digital model in lateral view. Scale bar, 100 mm. (b) Aquatic female northern elephant seal (*Hydrurga leptonyx*) skull. Top, dorsal view of CT scan; middle, sagittal section of skull from CT scan; bottom, 3D digital model in lateral view. Scale bar, 50 mm. The elephant seal skull is laterally (blue arrow) and ventrally (pink arrow) constricted in the ethmoid turbinal region relative to the polar bear.

Finally, the inverse relationship between RelCP and diving depth and duration may also indicate a structural constraint on CP size imposed by the stresses of diving. The adverse effects of diving to depth are well cited in the literature. Two notable effects are (a) the risk of increased nitrogen diffusion into the blood stream as gas tensions rise within air‐filled cavities and (b) the potential deformation of tissue surrounding compressed gas‐filled cavities (Kooyman & Ponganis, 1998). Adaptations to these challenges include, among others, collapsible alveoli, which minimize gas exchange (Scholander, 1940), distensible venous sinuses, which are thought to reduce external and middle ear cavity volume (Odend’hal & Poulter, 1966; Stenfors, Sadé, Hellström, & Anniko, 2001), and structural reductions in skull cavities, such as the narrowing of the external auditory canal (Kastak & Schusterman, 1999) and the loss of frontal sinuses (Curtis et al., 2015). Reductions in air‐filled skull cavities, while adaptive under hydrostatic pressure, may constrain the development of the olfactory recess in the aquatic mammal skull, specifically the olfactory turbinals and attendant airway fluid dynamics necessary for robust odorant deposition (Craven, Paterson, & Settles, 2009). In terrestrial carnivorans, ethmoturbinals often extend from the nasal cavity dorsally into the frontal sinuses, increasing surface area for odorant deposition and detection (Negus, 1958) (Figure A5a). By contrast, without the doming of the skull afforded by large frontal sinuses, the space available for ethmoturbinals and the CP in pinnipeds and the sea otter is limited dorsally (Figure A5b). Moreover, a survey of snout lengths in our sample reveals that aquatic carnivorans have significantly shorter snouts than terrestrial carnivorans, further reducing the nasal air space, and thereby perhaps constraining anterior extensions of ethmoturbinals as well (Tukey, *p = *.036, Table A4). Two exceptions to this are the California sea lion (*Zalophus californianus*) and the leopard seal (*Hydrurga leptonyx*), both of which have ethmoturbinals that extend into relatively long anterior nasal cavities, and large RelCPs as well (Figure 3 Appendix A1, Figure A5b). Finally, because aquatic carnivorans possess visual specializations for underwater vision, including relatively large eyeballs and orbits (Debey & Pyenson, 2013), the posterior nasal cavity is relatively narrow in most pinniped species (Berta et al., 2015), and most markedly in the elephant seals, further limiting space for ethmoturbinals laterally as well as ventrally (Figure A6a,b). Because ethmoturbinals and CP are developmentally linked and their surface areas tightly correlated (Bird et al., 2014), we expect any structural constraints on ethmoturbinal development to be reflected in smaller CPs as well. A future comparative study of ethmoturbinal surface area and nasal cavity volume across a large sample of aquatic carnivorans would be needed to test this (Van Valkenburgh et al., 2011). Additionally, to better resolve whether diving pressures have imposed adaptive structural constraints on the ethmoturbinal development in aquatic carnivorans, Finite Element Analysis (FEA) could be used to estimate the effects of variable compressive forces on bone surrounding air‐filled skull cavities, ethmoturbinal bones and the cribriform plate itself (Alam, Amini, Tadayon, Miserez, & Chinsamy, 2016).

Whereas many studies highlight the acquisition of multiple adaptations to aquatic life, we focused on a single loss, that is, reductions in the cribriform plate within two lineages that independently reinvaded marine environments, Pinnipedia, and Mustelidae. Among the mustelids, there were two parallel invasions of the water, one within the otters (Lutrinae) and the second within the weasels (Mustelinae) as represented by the mink. Relative to all the terrestrial arctoids in our sample except for the long‐tailed weasel, the mink and both otters have reduced RelCP that likely evolved in parallel. As each group (pinnipeds, mustelids) independently evolved to forage underwater, a central function of the olfactory apparatus, prey detection, became less important to diving carnivorans. Based on our results and established olfactory losses in cetaceans, we might expect reduced CP size to be a convergent adaptation among all marine mammals. To answer this, we need to investigate the cribriform plate morphology of mammals from all the lineages that secondarily invaded the sea, including the extant Afrotherian Sirenia as well as extinct aquatic mammals, such as the Afrotherian Desmostylia, Xenarthran *Thalassocnus* sloths (Amson, Billet, & de Muizon, 2018), stem Pinnipedia (*Enaliarctos* and *Puijila*), and stem cetaceans. Because the CP is fairly well preserved in the fossil record (Amson et al., 2018; Bird et al., 2018), it allows us to work backwards in deep time and visualize the evolutionary loss of olfactory anatomy among mammals as they transition from land to water. The cribriform plate, an informative, osseous record of olfactory activity in living and extinct mammals, offers a critical look into the evolution of olfaction at depth.

## CONFLICT OF INTEREST

The authors declare no conflict of interest.

## AUTHOR CONTRIBUTION

**Deborah Jean Bird:** Conceptualization (lead); Data curation (lead); Formal analysis (lead); Funding acquisition (supporting); Investigation (lead); Methodology (lead); Project administration (supporting); Resources (supporting); Supervision (lead); Writing‐original draft (lead); Writing‐review & editing (equal). **Iman Hamid:** Data curation (supporting); Formal analysis (supporting); Investigation (supporting); Methodology (supporting). **Lester Fox‐Rosales:** Data curation (supporting); Formal analysis (supporting); Investigation (supporting); Methodology (supporting). **Blaire Van Valkenburgh:** Conceptualization (equal); Formal analysis (equal); Funding acquisition (lead); Investigation (equal); Methodology (equal); Project administration (lead); Resources (lead); Supervision (supporting); Writing‐review & editing (equal). 

## Figures and Tables

**TABLE A3:**
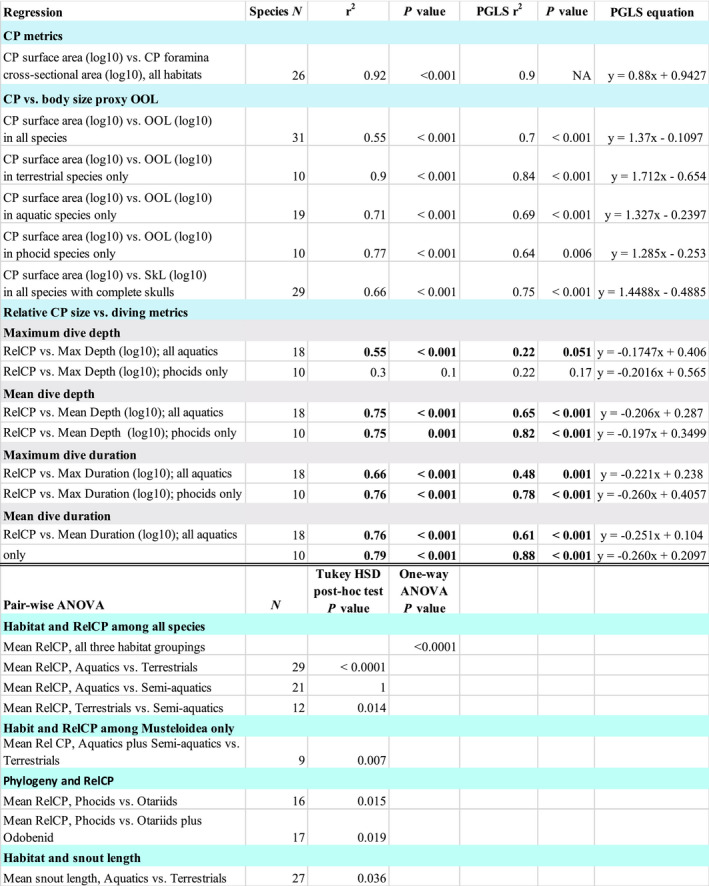
Summary statistics

Abbreviations: CP, cribriform plate; OOL, occipital condyle to orbit length; PGLS, phylogenetic generalized least squares regression; RelCP, relative cribriform plate size; SkL, skull length (occipital condyle to prosthion).

## Data Availability

Please see the Appendix for tables and figures. Within the text, we have referred readers to MorphoSource as well as Digimorph as open access repositories for computed tomography scan data as well as digital images. All files are available on Dryad: https://orcid.org/0000‐0001‐8217‐8985; https://doi.org/10.5068/D1CQ2G

## APPENDIX 1

### CONTACT FOR RESOURCE SHARING

Further information and requests for resources, scanning parameters, CT scan files, and 3D skull models should be directed to and will be fulfilled by the Lead Contact, Deborah Bird (dbirdseed@gmail.com).

**TABLE A1:** Study specimens, morphological data and scan data sources

Species	Common name	Sex	ID number	CP (mm^2^)	OOL (mm)	SkL (mm)	SnL (mm)	Habitat
*Mephitis mephitis*	Striped skunk	M	USNM147553	334.65	56.49	79.78	23.29	T
*Mephitis mephitis*	Striped skunk	F	UCLAJXS001	279.35	50.05	70.35	20.3	T
*Enhydra lutris*	Sea otter	M	SO2951	363.13	113.93	133.2	19.27	A
*Enhydra lutris*	Sea otter	F	SO2853‐97	321.03	106.29	126.94	20.65	A
*Gulo gulo*	Wolverine	M	USNM314885	889.6	103.6	173.36	69.76	T
*Gulo gulo*	Wolverine	F	USNM157327	813.88	100.78	152.5	51.72	T
*Lontra canadensis*	No. Amer. river otter	M	UCLA15275	223.29	87.14	121.93	34.79	SA
*Lontra canadensis*	No. Amer. river otter	F	UCLA18958	280.77	89.24	121.02	31.78	SA
*Mustela frenata*	Long tailed weasel	M	USNM52702	70.43	38.54	49.78	11.24	T
*Mustela frenata*	Long tailed weasel	F	USNM95054	49.5	31.55	40.27	8.72	T
*Neovison vison*	American mink	M	UCLA8488	125.09	59.96	73.51	13.55	SA
*Taxidea taxus*	American badger	M	UCLA14841	558.62	78	121.79	43.79	T
*Taxidea taxus*	American badger	F	LACM45012	644.17	86.3	128.99	42.69	T
*Potos flavus*	Kinkajou	M	USNM291066	268.54	66.76	88.71	21.95	T
*Potos flavus*	Kinkajou	F	LACM07241	228.84	64.56	87.45	22.89	T
*Procyon lotor*	Raccoon	M	LACM52261	353.08	75.53	120.95	45.42	T
*Procyon lotor*	Raccoon	F	LACM07241	412	77.94	122.62	44.68	T
*Ailuropda melanoleuca*	Giant panda	U	CAS6072	741.58	170.52	247.93	77.41	T
*Ursus americanus*	American black bear	M	USNM22070	2218.1	178.74	231.46	52.72	T
*Ursus americanus*	American black bear	F	USNM211397	1339.4	129.79	261.7	131.91	T
*Ursus americanus*	American black bear	M	MVZ162985	1607.4	196.6	301.2	104.6	T
*Ursus arctos*	Grizzly bear	M	USNM82003	2964.9	237.14	371.24	134.1	T
*Ursus arctos*	Grizzly bear	F	USNM98062	2317.9	205.65	325.7	120.05	T
*Ursus arctos*	Grizzly bear	U	MFWP113	1467.8	194.8	277.88	83.08	T
*Ursus maritimus*	Polar bear	F	MVZ123991	2453.6	232.1	329.2	97.1	T
*Ursus maritimus*	Polar bear	M	H001_51	2800.1	266.74	374.41	107.67	T
*Ursus maritimus*	Polar bear	U	USNM275072	3523.5	279.7	395.22	115.52	T
*Erignathus barbatus*	Bearded seal	M	LACM072575	337.6	139.56	195.57	56.01	A
*Erignathus barbatus*	Bearded seal	F	LACM072576	468.15	155.01	222.8	67.79	A
*Hydrurga leptonyx*	Leopard seal	M	USNM270326	992.41	254.23	374.49	120.26	A
*Hydrurga leptonyx*	Leopard seal	F	USNM269533	973.3	273.88	385.05	111.17	A
*Leptonychotes weddellii*	Weddell seal	F	MVZ127755	548.55	214.5	NA	NA	A
*Lobodon carcinophagus*	Crabeater seal	U	MVZ127751	552.23	175.53	246.13	70.6	A
*Lobodon carcinophagus*	Crabeater seal	F	MVZ127754	541.97	156.11	218.79	62.68	A
*Mirounga angustirostris*	No. elephant seal	F	MVZ 184140	310.62	181.51	241.74	60.23	A
*Mirounga angustirostris*	No. elephant seal	M	LACM054394	729	352.92	481.74	128.82	A
*Mirounga leonina*	So. elephant seal	M	LACM084290	755.24	365	NA	NA	A
*Mirounga leonina*	So. elephant seal	F	LACM084245	575	264.93	NA	NA	A
*Neomonachus schauinslandi*	Hawaiian monk seal	U	LACM54438	453.85	184.95	NA	NA	A
*Neomonachus schauinslandi*	Hawaiian monk seal	U	LACM53325	451.18	176.64	230.72	54.08	A
*Neomonachus tropicalis*	Tropical monk seal	M	USNM100358	510.89	187.09	268.61	81.52	A
*Neomonachus tropicalis*	Tropical monk seal	F	USNM102527	407	170.38	236.7	66.32	A
*Pusa sibirica*	Baikal seal	U	LACM52337	187.99	109.44	150.04	40.6	A
*Phoca vitulina*	Harbor seal	U	UCLA1408	339.3	156.9	NA	NA	A
*Phoca vitulina*	Harbor seal	M	LACM095963	295	125.21	167.61	42.4	A
*Phoca vitulina*	Harbor seal	F	LACM31462	348.67	139.04	199.53	60.49	A
*Pusa hispida*	Ringed seal	M	LACM54781	216.29	132.37	177.75	45.38	A
*Pusa hispida*	Ringed seal	F	LACM22949	195.35	115.75	152.01	36.26	A
*Arctocephalus galapagoensis*	Galapagos fur seal	M	LACM031309	524.4	152.22	199.69	47.47	A
*Arctocephalus pusillus*	Cape fur seal	M	LACM052358	614.92	190.35	252.9	62.55	A
*Arctocephalus pusillus*	Cape fur seal	F	LACM052359	396.92	155.49	196.77	41.28	A
*Callorhinus ursinus*	Northern fur seal	M	LACM052343	453.18	144.56	194.96	50.4	A
*Callorhinus ursinus*	Northern fur seal	F	LACM054630	411.63	149.01	188.51	39.5	A
*Eumetopias jubatus*	Steller sea lion	M	LACM052314	1475.2	295.39	374.75	79.36	A
*Eumetopias jubatus*	Steller sea lion	F	LACM052316	996.41	241.81	310.61	68.8	A
*Otaria flavescens*	So. Amer. sea lion	M	LACM095756	681.19	237.95	335.19	97.24	A
*Otaria flavescens*	So. Amer. sea lion	F	LACM095771	461.62	161.96	208.94	46.98	A
*Zalophus californianus*	California sea lion	M	UCLA252	868.25	214.3	291.75	77.45	A
*Zalophus californianus*	California sea lion	F	LACM95730	599.49	155.72	203.29	47.57	A
*Zalophus californianus*	California sea lion	M	UCLA1118	888.52	200.39	NA	NA	A
*Odobenus rosmarus*	Walrus	F	UCLA2471	1018.2	258.82	350.42	91.6	A
*Odobenus rosmarus*	Walrus	M	UCLA15306	1104.7	270.46	367.47	97.01	A

High resolution CT scanner	Scanning facility
GE Phoenix nanotom s	Molecular Imaging Center, University of Southern California
Nikon Metrology XT H 225 ST	Molecular Imaging Center, University of Southern California
North Star Imaging ACTIS	The Univ. of Texas High‐Resolution X‐ray Computed Tomography Facility
Xradia microXCT	The Univ. of Texas High‐Resolution X‐ray Computed Tomography Facility
Siemens SOMATOM definition AS64	Ronald Reagan Medical Center UCLA
Imaging software	
Mimics v. 15.0‐21.0	Materialise; Leuven, Belgium
3‐Matics v. 7.0.1‐13.0	Materialise; Leuven, Belgium
Rhinoceros v. 4	Robert McNeel and Associates

Abbreviations: A, Aquatic; CP, cribriform plate surface area; F, female; M, male; OOL, occiput‐orbit length (occipital condyle to prosthion); SA, Semi‐aquatic; SkL, Skull length (occipital condyle to prosthion); SnL, Snout length (anterior orbit border to prosthion); T, Terrestrial; U, unknown sex.

**TABLE A2:** Dive data for all aquatic species, and sources. Maximum dive depth and duration values were sourced from single studies. Mean dive depth and duration were sourced and averaged from multiple studies where available. In calculating an overall average, individual study averages were weighted by sample size.

Species	Mean depth (m)	*N*	References	Mean duration (min)	*N*	References	Max. depth (m)	References	Max. duration (min)	References
Phocidae										
*Erignathus barbatus*	20.98	11	Krafft, Lydersen, Kovacs, Gjertz, and Haug (2000), Hamilton, Kovacs, and Lydersen (2018)	3.73	11	Krafft et al. (2000) and Hamilton et al. (2018)	472	Gjertz, Kovacs, Lydersen, and Wiig (2000)	24	Hamilton et al. (2018)
*Hydrurga leptonyx*	17.12	29	Kuhn et al. (2006), Krause, Goebel, Marshall, and Abernathy (2015) and Krause, Goebel, Marshall, and Abernathy (2016)	2.19	22	Kuhn et al. (2006) and Krause et al. (2016)	424.5	Kuhn et al. (2006)	15	Nordøy and Blix (2008)
*Leptonychotes weddellii*	163.21	43	Schreer and Testa (1996), Costa, Huckstadt, et al. (2010), Costa, Robinson, et al. (2010), Heerah et al. (2013) and Shero, Goetz, Costa, and Burns (2018)	13.24	43	Schreer and Testa (1996), Costa, Robinson, et al. (2010), Costa, Huckstadt, et al. (2010), Heerah et al. (2013) and Shero et al. (2018)	904	Heerah et al. (2013)	96	Heerah et al. (2013)
*Lobodon carcinophagus*	47.97	80	Costa, Huckstadt, et al. (2010), Bengtson and Stewart (1992), Nordøy, Folkow, and Blix (1995), Burns et al. (2004) and Wall, Bradshaw, Southwell, Gales, and Hindell (2007)	3.02	74	Costa, Huckstadt, et al. (2010), Burns et al. (2004), Wall et al.(2007) and Nordøy et al. (1995)	712.5	Burns et al. (2004)	23.6	Burns et al. (2004)
*Mirounga angustirostris*	504.76	353	LeBoeuf, Costa, Huntley, and Feldkamp (1989), Delong and Stewart (1991, 2005), Stewart and Delong (1995), Robinson et al. (2012) and Naito et al. (2013)	22.997	353	LeBoeuf et al. (1989), Delong and Stewart (1991, 2005), Stewart and Delong (1995), Robinson et al. (2012) and Naito et al. (2013)	1735	Robinson et al. (2012)	119	Stewart & Delong, 1995)
*Mirounga leonina*	443.9	136	Costa, Huckstadt, et al. (2010), Hindell, Slip, and Burton (1991), Campagna, Le Boeuf, Blackwell, Crocker, and Quintana (1995), Bennett (2001), McIntyre et al. (2010), Thums, Bradshaw, Sumner, Horsburgh, and Hindell (2013) and Muelbert, de Souza, Lewis, and Hindell (2013)	28.424	136	Costa, Huckstadt, et al. (2010), Hindell et al. (1991), Campagna et al. (1995), Bennett (2001), McIntyre et al. (2010), Thums et al. (2013), and Muelbert et al. (2013)	2388	Costa, Robinson, et al., 2010; Costa, Huckstadt, et al., 2010)	120	Hindell et al. (1991)
*Neomonachus schauinslandi*	33.822	48	Parrish, Abernathy, Marshall, and Buhleier (2002) pers. comm.	8	24	Parrish, Craig, Ragen, Marshall, and Buhleier (2000)	500	Parrish, Abernathy, Marshall, and Buhleier (2002)	20	Parrish et al. (2002)
*Pusa sibirica*	83.356	9	Watanabe, Baranov, Sato, Naito, and Miyazaki (2004), Watanabe (2006) and Watanabe, Baranov, and Miyazaki (2015)	7.81	9	Watanabe (2006), Watanabe et al. (2015) and Watanabe et al. (2004)	324	Watanabe (2006)	>40	Stewart, Petrov, Baranov, Timonin, and Ivanov (1996)
*Phoca vitulina*	56.81	56	Gjertz, Lydersen, and Wiig (2001), Eguchi, Harvey, and Harvey (2005) and Blanchet, Lydersen, Ims, and Kovacs (2015)	4.57	56	Gjertz et al. (2001), Blanchet et al. (2015) and Eguchi and Harvey (2005)	558	Kolb and Norris (1982)	37	Blanchet et al. (2015)
*Pusa hispida*	80.195	61	Gjertz, Kovacs, Lydersen, and Wiig (2000), Benoit, Simard, Gagné, Geoffroy, and Fortier, (2010) pers. comm., Harwood, Smith, Auld, Melling, and Yurkowski et al. (2016) pers. comm.	5.208	52	Gjertz et al. (2000)	542	Harwood et al. (2015)	>50	Gjertz et al. (2000)
Otariidae										
*Arctocephalus galapagoensis*	29.66	40	Kooyman and Trillmich (1986); Villegas‐Amtmann, Jeglinski, Costa, Robinson, and Trillmich, (2013) and Jeglinski, Goetz, Werner, Costa, and Trillmich (2013)	1.7	36	Villegas‐Amtmann et al. (2013) and Jeglinski et al. (2013)	131	Villegas‐Amtmann et al. (2013)	7.7	Kooyman and Trillmich (1986) (2014)
*Arctocephalus pusillus*	45	2	Kooyman and Gentry (1986)	2.1	2	Kooyman et al. (1986, 2006)	204	Kooyman et al. (1986, 2006)	7.5	Kooyman et al. (1986, 2006)
*Callorhinus ursinus*	26.383	111	Goebel, Bengtson, Delong, Gentry, and Loughlin, (1991), Sterling and Ream (2004), Kuhn (2011) and Skinner, Burkanov, and Andrews (2012)	1.291	61	Sterling and Ream (2004), Kuhn (2011) and Skinner et al. (2012)	256	Gentry, Kooyman, and Goebel (1986)	9.92	Sterling and Ream (2004)
*Eumetopias jubatus*	35.522	18	Merrick, Loughlin, Antonelis, and Hill (1994), Merrick and Loughlin (1997) and Loughlin, Perlov, Baker, Blokhin, and Makhnyr (1998)	1.786	18	Merrick et al. (1994), Merrick and Loughlin (1997) and Loughlin et al. (1998)	328	Loughlin, Sterling, Merrick, Sease, and York (2003)	14	Loughlin et al. (2003)
*Otaria flavescens*	42.207	67	Werner & Campagna (1995), Thompson et al. (1998), Baylis et al. (2015), Hückstädt et al. (2016	1.95	67	Werner & Campagna (1995), Thompson et al. (1998), Baylis et al. (2015), Hückstädt et al., (2016)	325	Hückstädt et al. (2016)	12.07	Hückstädt et al. (2016)
*Zalophus californianus*	44.28	127	Costa et al. (2007) and Thomas, Harvey, Goldstein, Barakos, and Gulland (2010)	1.71	127	Costa et al. (2007), Thomas et al. (2010)	575	Costa et al. (2007)	20.1	Costa et al. (2007)
Odobenidae										
*Odobenus rosmarus*	15.4	8	Gjertz, Griffiths, Krafft, Lydersen & Wiig (2001), Wiig, Gjertz, Griffiths, and Lydersen (1993) and Nyholm (1975)	5.1	16	Nyholm, 1975; Gjertz et al. 2001; Born & Knutsen, 1997)	100	Fay and Burns (1988)	24	Gjertz et al. (2001)
Mustelidae										
*Enhydra lutris*	12.07	27	Bodkin et al. (2004) and Tinker et al. (2007)	1.28	27	Bodkin et al., 2004; Tinker et al., 2007; Ralls, Hatfield, & Siniff, 1995)	100	Bodkin et al. (2004)	4.1	Ralls et al. (1995)

## References

[ece36343-bib-0001] Alam, P. , Amini, S. , Tadayon, M. , Miserez, A. , & Chinsamy, A. (2016). Properties and architecture of the sperm whale skull amphitheatre. Zoology, 119, 42–51. 10.1016/j.zool.2015.12.001 26781232

[ece36343-bib-0002] Amson, E. , Billet, G. , & de Muizon, C. (2018). Evolutionary adaptation to aquatic lifestyle in extinct sloths can lead to systemic alteration of bone structure. Proceedings of the Royal Society B: Biological Sciences, 285, 20180270 10.1098/rspb.2018.0270 PMC5966604 29743254

[ece36343-bib-0003] Barnard, W. R. , Andreae, M. O. , Watkins, W. E. , Bingemer, H. , & Georgii, H. W. (1982). The flux of dimethylsulfide from the oceans to the atmosphere (Atlantic). Journal of Geophysical Research, 87, 8787–8793. 10.1029/JC087iC11p08787

[ece36343-bib-0004] Baylis, A. M. M. , Orben, R. A. , Arnould, J. P. Y. , Peters, K. , Knox, T. , Costa, D. P. , & Staniland, I. J. (2015). Diving deeper into individual foraging specializations of a large marine predator, the southern sea lion. Oecologia, 179, 1053–1065. 10.1007/s00442-015-3421-4 26323982

[ece36343-bib-0005] Beichman, A. C. , Koepfli, K.‐P. , Li, G. , Murphy, W. , Dobrynin, P. , Kliver, S. , … Wayne, R. K. (2019). Aquatic adaptation and depleted diversity: A Deep dive into the genomes of the sea otter and giant otter article fast track. Molecular Biology and Evolution, 36, 2631–2655. 10.1093/molbev/msz101 31212313 PMC7967881

[ece36343-bib-0006] Bengtson, J. L. , & Stewart, B. S. (1992). Diving and haulout behavior of crabeater seals in the Weddell Sea, Antarctica, during March 1986. Polar Biology, 12, 635–644. 10.1007/BF00236986

[ece36343-bib-0007] Bennett, K. A. (2001). Diurnal and seasonal variations in the duration and depth of the longest dives in southern elephant seals (*Mirounga leonina*): Possible physiological and behavioral constraints. The Journal of Experimental Biology, 204, 649–662. 11171347 10.1242/jeb.204.4.649

[ece36343-bib-0008] Benoit, D. , Simard, Y. , Gagné, J. , Geoffroy, M. , & Fortier, L. (2010). From polar night to midnight sun: Photoperiod, seal predation, and the diel vertical migrations of polar cod (*Boreogadus saida*) under landfast ice in the Arctic Ocean. Polar Biology, 33, 1505–1520. 10.1007/s00300-010-0840-x

[ece36343-bib-0009] Berta, A. , Sumich, J. L. , & Kovacs, K. M. (2015). Marine mammals: Evolutionary biology. Burlington, MA: Academic Press.

[ece36343-bib-0010] Bird, D. J. , Amirkhanian, A. , Pang, B. , & Van Valkenburgh, B. (2014). Quantifying the cribriform plate: Influences of allometry, function, and phylogeny in Carnivora. The Anatomical Record, 297, 2080–2092. 25312366 10.1002/ar.23032

[ece36343-bib-0011] Bird, D. J. , Murphy, W. J. , Fox‐Rosales, L. , Hamid, I. , Eagle, R. A. , & Van Valkenburgh, B. (2018). Olfaction written in bone: Cribriform plate size parallels olfactory receptor gene repertoires in Mammalia. Proceedings of the Royal Society B: Biological Sciences, 285, 1–9. 10.1098/rspb.2018.0100 PMC5879636 29540522

[ece36343-bib-0012] Blanchet, M. A. , Lydersen, C. , Ims, R. A. , & Kovacs, K. M. (2015). Seasonal, oceanographic and atmospheric drivers of diving behaviour in a temperate seal species living in the high arctic. PLoS One, 10, 1–28. 10.1371/journal.pone.0132686 PMC4509669 26196289

[ece36343-bib-0013] Bodkin, J. L. , Esslinger, G. G. , & Monson, D. H. (2004). Foraging depth of sea otters and implications to coastal marine communities. Marine Mammal Science, 20, 305–321. 10.1111/j.1748-7692.2004.tb01159.x

[ece36343-bib-0014] Born, E. W. , & Knutsen, L. (1997). Haul‐out and diving activity of male atlantic walruses (Odobenus rosmarus rosmarus) in NE Greenland. Journal of Zoology, 243, 381–396. 10.1111/j.1469-7998.1997.tb02789.x

[ece36343-bib-0015] Bouchard, B. , Barnagaud, J. , Poupard, M. , Gauffier, P. , Ortiz, S. , Lisney, T. J. , … Rasmussen, M. (2019). Behavioural responses of humpback whales to food‐related chemical stimuli. PLoS One, 14, 1–23. 10.1371/journal.pone.0212515 PMC6391047 30807595

[ece36343-bib-0017] Bowen, W. D. , & Siniff, D. B. (1999). Distribution, population biology, and feeding ecology of marine mammals In Reynolds J. E. III, & Rommel S. A. (Eds.), Biology of marine mammals (pp. 423–484). Washington, DC: Smithsonian Institution Press.

[ece36343-bib-0018] Burns, J. M. , Costa, D. P. , Fedak, M. A. , Hindell, M. A. , Bradshaw, C. J. A. , Gales, N. J. , … Crocker, D. E. (2004). Winter habitat use and foraging behavior of crabeater seals along the Western Antarctic Peninsula. Deep Sea Research Part II: Topical Studies in Oceanography, 51, 2279–2303. 10.1016/j.dsr2.2004.07.021

[ece36343-bib-0019] Campagna, C. , Le Boeuf, B. J. , Blackwell, S. B. , Crocker, D. E. , & Quintana, F. (1995). Diving behaviour and foraging location of female southern elephant seals from Patagonia. Journal of Zoology, 236, 55–71. 10.1111/j.1469-7998.1995.tb01784.x

[ece36343-bib-0020] Catania, K. C. , Hare, J. F. , & Campbell, K. L. (2008). Water shrews detect movement, shape, and smell to find prey underwater. Proceedings of the National Academy of Sciences of the United States of America, 105, 571–576. 18184804 10.1073/pnas.0709534104 PMC2206577

[ece36343-bib-0021] Costa, D. P. , Huckstadt, L. A. , Crocker, D. E. , Mcdonald, B. I. , & Michael, E. (2010). Approaches to studying climatic change and its role on the habitat selection of Antarctic Pinnipeds. Integrative and Comparative Biology, 50, 1018–1030. 10.1093/icb/icq054 21558256

[ece36343-bib-0022] Costa, D. P. , Kuhn, C. , & Weise, M. (2007). Foraging ecology of the California sea lion: Diet, diving behavior, foraging locations, and predation impacts on fisheries resources. UC San Diego: California Sea Grant College Program. .Retrieved from https://escholarship.org/uc/item/9gr5784d

[ece36343-bib-0023] Costa, D. P. , Robinson, P. W. , Arnould, J. P. Y. , Harrison, A.‐L. , Simmons, S. E. , Hassrick, J. L. , … Crocker, D. E. (2010). Accuracy of ARGOS locations of pinnipeds at‐sea estimated using Fastloc GPS. PLoS One, 5, e8677 10.1371/journal.pone.0008677 20090942 PMC2806907

[ece36343-bib-0024] Craven, B. A. , Paterson, E. G. , & Settles, G. S. (2009). The fluid dynamics of canine olfaction: Unique nasal airflow patterns as an explanation of macrosmia. Journal of the Royal Society Interface, 7, 933–943. 10.1098/rsif.2009.0490 PMC2871809 20007171

[ece36343-bib-0025] Cunningham, L. , Baxter, J. M. , Boyd, I. L. , Duck, C. D. , Lonergan, M. , Moss, S. E. , & McConnell, B. (2009). Harbour seal movements and haul‐out patterns: Implications for monitoring and management. Aquatic Conservation: Marine and Freshwater Ecosystems, 19, 398–407. 10.1002/aqc.983

[ece36343-bib-0026] Curtis, A. A. , Lai, G. , Wei, F. , & Van Valkenburgh, B. (2015). Repeated loss of frontal sinuses in arctoid carnivorans. Journal of Morphology, 276, 22–32. 10.1002/jmor.20313 25069818

[ece36343-bib-0027] Dacey, J. W. H. , & Wakeham, S. G. (1986). Oceanic dimethylsulfide: Production during zooplankton grazing on phytoplankton. Science, 233, 1314–1316. 10.1126/science.233.4770.1314 17843360

[ece36343-bib-0028] Davis, R. W. , Fuiman, L. A. , Williams, T. M. , Horning, M. , & Hagey, W. (2003). Classification of Weddell seal dives based on 3‐dimensional movements and video‐recorded observations. Marine Ecology Progress Series, 264, 109–122.

[ece36343-bib-0029] Davis, R. W. , Fuiman, L. A. , Williams, T. M. , & Le Boeuf, B. J. (2001). Three‐dimensional movements and swimming activity of a northern elephant seal. Comparative Biochemistry and Physiology Part A: Molecular & Integrative Physiology, 129, 759–770. 10.1016/S1095-6433(01)00345-2 11440863

[ece36343-bib-0030] Debey, L. B. , & Pyenson, N. D. (2013). Osteological correlates and phylogenetic analysis of deep diving in living and extinct pinnipeds: What good are big eyes? Marine Mammal Science, 29, 48–83. 10.1111/j.1748-7692.2011.00545.x

[ece36343-bib-0031] Dehnhardt, G. , Mauck, B. , & Bleckmann, H. (1998). Seal whiskers detect water movements. Nature, 394, 235–236. 10.1038/28303

[ece36343-bib-0032] DeLong, R. L. , Kooyman, G. L. , Gilmartin, W. G. , & Loughlin, T. R. (1984). Hawaiian monk seal diving behavior. Acta Zoologica Fennica, 172, 129–131.

[ece36343-bib-0033] Delong, R. L. , & Stewart, B. S. (1991). Diving patterns of northern elephant seal bulls. Marine Mammal Science, 7, 369–384. 10.1111/J.1748-7692.1991.Tb00112.X

[ece36343-bib-0034] Eguchi, T. , & Harvey, J. (2005). Diving behavior of the Pacific harbor seal (*Phoca vitulina richardii*) in Monterey Bay, California. Marine Mammal Science, 21, 283–295. 10.1111/j.1748-7692.2005.tb01228.x

[ece36343-bib-0036] Fay, F. H. , & Burns, J. J. (1988). Maximal feeding depth of Walruses. Arctic, 41(3), 239–240. 10.14430/arctic1724

[ece36343-bib-0038] Gentry, R. L. , Kooyman, G. L. , & Goebel, M. E. (1986). Feeding and diving behavior in Northern Fur Seals In Gentry R. L., & Kooyman G. L. (Eds.), Fur seals: Maternal strategies on land and at sea (pp. 61–78). Princeton, NJ: Princeton University Press.

[ece36343-bib-0039] Gittleman, J. L. (2013). Carnivore behavior, ecology, and evolution. Dordrecht, NL: Springer Science & Business Media.

[ece36343-bib-0040] Gjertz, I. , Griffiths, D. , Krafft, B. A. , Lydersen, C. , & Wiig, Ø. (2001). Diving and haul‐out patterns of walruses *Odobenus rosmarus* on Svalbard. Polar Biology, 24, 314–319. 10.1007/s003000000211

[ece36343-bib-0041] Gjertz, I. , Kovacs, K. M. , Lydersen, C. , & Wiig, Ø. (2000). Movements and diving of bearded seal (*Erignathus barbatus*) mothers and pups during lactation and post‐weaning. Polar Biology, 23, 559–566. 10.1007/s003000000121

[ece36343-bib-0042] Gjertz, I. , Kovacs, K. M. , Lydersen, C. , & Wiig, Ø. (2000). Movements and diving of adult ringed seals (*Phoca hispida*) in Svalbard. Polar Biology, 23, 651–656. 10.1007/s003000000143

[ece36343-bib-0043] Gjertz, I. , Lydersen, C. , & Wiig, Ä. (2001). Distribution and diving of harbour seals (*Phoca vitulina*) in Svalbard. Polar Biology, 24, 209–214. 10.1007/s003000000197

[ece36343-bib-0044] Goebel, M. E. , Bengtson, J. L. , Delong, R. L. , Gentry, R. L. , & Loughlin, T. R. (1991). Diving patterns and foraging locations of female northern fur seals. Fishery Bulletin, 89, 171–179.

[ece36343-bib-0045] Graziadei, P. P. C. , & Graziadei, G. A. M. (1985). Neurogenesis and plasticity of the olfactory sensory neurons. Annals of the New York Academy of Sciences, 457, 127–142. 10.1111/j.1749-6632.1985.tb20802.x 3913359

[ece36343-bib-0046] Hamilton, C. D. , Kovacs, K. M. , & Lydersen, C. (2018). Individual variability in diving, movement and activity patterns of adult bearded seals in Svalbard, Norway. Scientific Reports, 8, 1–17. 10.1038/s41598-018-35306-6. 30451906 PMC6242851

[ece36343-bib-0047] Harcourt, R. G. , Hindell, M. A. , & Bell, D. G. (2000). Three‐dimensional dive profiles of free‐ranging Weddell seals. Polar Biology, 23, 479–487.

[ece36343-bib-0048] Harrison, R. J. , & Kooyman, G. L. (1968). General physiology of the pinnipedia In Harrison R. J., Hubbard R. C., Peterson R. S., Rice C. E., & Schusterman R. J. (Eds.), The behavior and physiology of pinnipeds (pp. 211–296). New York, NY: Appleton‐Century‐Crofts.

[ece36343-bib-0049] Harwood, L. A. , Smith, T. G. , Auld, J. C. , Melling, H. , & Yurkowski, D. J. (2015). Seasonal movements and diving of ringed seals, *Pusa hispida*, in the western canadian arctic, 1999–2001 and 2010–11. Arctic, 68, 193–209. 10.14430/arctic4479

[ece36343-bib-0050] Hayden, S. , Bekaert, M. , Crider, T. A. , Mariani, S. , Murphy, W. J. , & Teeling, E. C. (2010). Ecological adaptation determines functional mammalian olfactory subgenomes. Genome Research, 20, 1–9. 10.1101/gr.099416.109 19952139 PMC2798820

[ece36343-bib-0051] Heerah, K. , Andrews‐Goff, V. , Williams, G. , Sultan, E. , Hindell, M. , Patterson, T. , & Charrassin, J. B. (2013). Ecology of Weddell seals during winter: Influence of environmental parameters on their foraging behaviour. Deep Sea Research Part II: Topical Studies in Oceanography, 88–89, 23–33. 10.1016/j.dsr2.2012.08.025

[ece36343-bib-0052] Hindell, M. A. , Slip, D. J. , & Burton, H. R. (1991). The diving behaviour of adult male and female southern elephant seals, *Mirounga leonina* (Pinnipedia: Phocidae). Australian Journal of Zoology, 39, 499–508. 10.1071/ZO9910595

[ece36343-bib-0053] Hückstädt, L. A. , Tift, M. S. , Riet‐Sapriza, F. , Franco‐Trecu, V. , Baylis, A. M. M. , Orben, R. A. , … Costa, D. P. (2016). Regional variability in diving physiology and behavior in a widely distributed air‐breathing marine predator, the South American sea lion (*Otaria byronia*). The Journal of Experimental Biology, 219, 2320–2330. 10.1242/jeb.138677 27247316

[ece36343-bib-0054] Hughes, G. M. , Gang, L. , Murphy, W. J. , Higgins, D. G. , & Teeling, E. C. (2013). Using Illumina next generation sequencing technologies to sequence multigene families in de novo species. Molecular Ecology Resources, 13, 510–521. 10.1111/1755-0998.12087 23480365

[ece36343-bib-0016] Irvine, L. G. , Hindell, M. A. , Van Hoff, J. , & Burton, H. R. (2000). The influence of body size on dive duration of underyearling southern elephant seals (*Mirounga leonina*). Journal of Zoology, 251, 463–471. 10.1017/S0952836900008062

[ece36343-bib-0055] Jeglinski, J. W. E. , Goetz, K. T. , Werner, C. , Costa, D. P. , & Trillmich, F. (2013). Same size ‐ same niche? Foraging niche separation between sympatric juvenile Galapagos sea lions and adult Galapagos fur seals. The Journal of Animal Ecology, 82, 694–706. 10.1111/1365-2656.12019 23351022

[ece36343-bib-0056] Kastak, D. , & Schusterman, R. J. (1999). In‐air and underwater hearing sensitivity of a northern elephant seal (*Mirounga angustirostris*). Canadian Journal of Zoology, 77, 1751–1758. 10.1139/cjz-77-11-1751

[ece36343-bib-0057] King, J. E. (1983). Seals of the world.London, UK: British Museum (Natural History)

[ece36343-bib-0058] Kishida, T. , Thewissen, J. G. M. , Hayakawa, T. , Imai, H. , & Agata, K. (2015). Aquatic adaptation and the evolution of smell and taste in whales. Zoological Letters, 1, 9 10.1186/s40851-014-0002-z 26605054 PMC4604112

[ece36343-bib-0059] Kolb, P. M. , & Norris, K. S. (1982). A harbor seal, Phoca vitulina richardsi, taken from a sablefish trap. California Fish and Game, 68, 123–124.

[ece36343-bib-0060] Kooyman, G. L. (1973). Respiratory adaptations in marine mammals. Integrative and Comparative Biology, 13, 457–468. 10.1093/icb/13.2.457

[ece36343-bib-0061] Kooyman, G. L. , & Gentry, R. L. (1986). Diving behavior of South African fur seals In Gentry R. L., & Kooyman G. L. (Eds.), Fur seals: Maternal strategies on land and at sea (pp. 142–152). Princeton, NJ: Princeton University Press.

[ece36343-bib-0062] Kooyman, G. L. , & Ponganis, P. J. (1998). The physiological basis of diving to depth: Birds and mammals. Annual Review of Physiology, 60, 19–32. 10.1146/annurev.physiol.60.1.19 9558452

[ece36343-bib-0037] Kooyman, G. L. , & Trillmich, F. (1986). Diving behavior of Galapagos fur seals In Gentry R. L., & Kooyman G. L. (Eds.), Fur seals: Maternal strategies on land and at sea (pp. 168–185). Princeton, NJ: Princeton University Press.

[ece36343-bib-0063] Kowalewsky, S. , Dambach, M. , Mauck, B. , & Dehnhardt, G. (2006). High olfactory sensitivity for dimethyl sulphide in harbour seals. Biology Letters, 2, 106–109. 10.1098/rsbl.2005.0380 17148339 PMC1617201

[ece36343-bib-0064] Krafft, B. A. , Lydersen, C. , Kovacs, K. M. , Gjertz, I. , & Haug, T. (2000). Diving behaviour of lactating bearded seals (*Erignathus barbatus*) in the Svalbard area. Canadian Journal of Zoology, 78, 1408–1418. 10.1139/cjz-78-8-1408

[ece36343-bib-0065] Krause, D. J. , Goebel, M. E. , Marshall, G. J. , & Abernathy, K. (2015). Novel foraging strategies observed in a growing leopard seal (*Hydrurga leptonyx*) population at Livingston Island, Antarctic Peninsula. Animal Biotelemetry, 3, 1–14. 10.1186/s40317-015-0059-2

[ece36343-bib-0066] Krause, D. J. , Goebel, M. E. , Marshall, G. J. , & Abernathy, K. (2016). Summer diving and haul‐out behavior of leopard seals (*Hydrurga leptonyx*) near mesopredator breeding colonies at Livingston Island, Antarctic Peninsula. Marine Mammal Science, 32, 839–867. 10.1111/mms.12309

[ece36343-bib-0067] Kröger, R. H. H. , & Katzir, G. (2008). Comparative anatomy and physiology of vision in aquatic tetrapods In Thewissen J.G.M. & Nummela S.(Eds.), Sensory evolution on the threshold: Adaptations in secondarily aquatic vertebrates (pp.121‐148). Berkeley, CA: University of California Press 10.1525/california/9780520252783.003.0009

[ece36343-bib-0068] Kuhn, C. E. (2011). The influence of subsurface thermal structure on the diving behavior of northern fur seals (*Callorhinus ursinus*) during the breeding season. Marine Biology, 158, 649–663. 10.1007/s00227-010-1589-z

[ece36343-bib-0069] Kuhn, C. E. , McDonald, B. I. , Shaffer, S. A. , Barnes, J. , Crocker, D. E. , Burns, J. , & Costa, D. P. (2006). Diving physiology and winter foraging behavior of a juvenile leopard seal (*Hydrurga leptonyx*). Polar Biology, 29, 303–307. 10.1007/s00300-005-0053-x

[ece36343-bib-0070] LeBoeuf, B. J. , Costa, D. P. , Huntley, A. C. , & Feldkamp, S. D. (1989). Continuous, deep diving in female northern elephant seals, *Mirounga angustirostris* . Canadian Journal of Zoology, 66, 446–458. 10.1139/z88-064

[ece36343-bib-0071] Levenson, D. H. , & Schusterman, R. J. (1999). Dark adaptation and visual sensitivity in shallow and deep‐diving pinnipeds. Marine Mammal Science, 15, 1303–1313. 10.1111/j.1748-7692.1999.tb00892.x

[ece36343-bib-0072] Liu, A. , He, F. , Shen, L. , Liu, R. , Wang, Z. , & Zhou, J. (2019). Convergent degeneration of olfactory receptor gene repertoires in marine mammals. BMC Genomics, 20, 1–14. 10.1186/s12864-019-6290-0 31842731 PMC6916060

[ece36343-bib-0073] Loughlin, T. R. , Perlov, A. S. , Baker, J. D. , Blokhin, S. A. , & Makhnyr, A. G. (1998). Diving behavior of adult female Steller sea lions in the Kuril Islands, Russia. Biosphere Conservation: for Nature, Wildlife, and Humans, 1, 21–31. 10.20798/biospherecons.1.1_21

[ece36343-bib-0074] Loughlin, T. R. , Sterling, J. T. , Merrick, R. L. , Sease, J. L. , & York, A. E. (2003). Diving behavior of immature Steller sea lions (*Eumetopias jubatus*). Fishery Bulletin, 101, 566–582.

[ece36343-bib-0075] Lowell, W. R. , & Flanigan, W. F. (1980). Marine mammal chemoreception. Mammal Review, 10, 53–59.

[ece36343-bib-0076] Marriott, A. , Robert, M. , Marriott, S. , Cowan, E. , Cohen, J. , & Hallock, R. M. (2013). Sniffing: Adaptations allow mammals without traditional olfactory capabilities to forage for food underwater capabilities to forage for food underwater. Zoological Science, 30, 69–75. 10.2108/zsj.30.69 23387839

[ece36343-bib-0077] Marshall, C. D. , Amin, H. , Kovacs, K. M. , & Lydersen, C. (2006). Microstructure and innervation of the mystacial vibrissal follicle‐sinus complex in bearded seals, *Erignathus barbatus* (Pinnipedia: Phocidae). The Anatomical Record Part A: Discoveries in Molecular, Cellular, and Evolutionary Biology, 288A, 13–25. 10.1002/ar.a.20273 16342212

[ece36343-bib-0078] McIntyre, T. , de Bruyn, P. J. N. , Ansorge, I. J. , Bester, M. N. , Bornemann, H. , Plötz, J. , & Tosh, C. A. (2010). A lifetime at depth: Vertical distribution of southern elephant seals in the water column. Polar Biology, 33, 1037–1048. 10.1007/s00300-010-0782-3

[ece36343-bib-0079] Merrick, R. L. , & Loughlin, T. R. (1997). Foraging behavior of adult female and young‐of‐the‐year Steller sea lions in Alaskan waters. Canadian Journal of Zoology, 75, 776–786. 10.1139/z97-099

[ece36343-bib-0080] Merrick, R. L. , Loughlin, T. R. , Antonelis, G. A. , & Hill, R. (1994). Use of satellite‐linked telemetry to study steller sea lion and northern fur seal foraging (*Eumetopias jubatus*, *Callorhinus ursinus*). Polar Research, 13(1), 105–114.

[ece36343-bib-0081] Muelbert, M. M. C. , de Souza, R. B. , Lewis, M. N. , & Hindell, M. A. (2013). Foraging habitats of southern elephant seals, *Mirounga leonina*, from the Northern Antarctic Peninsula. Deep Sea Research Part II: Topical Studies in Oceanography, 88–89, 47–60. 10.1016/j.dsr2.2012.07.009

[ece36343-bib-0082] Naito, Y. , Costa, D. P. , Adachi, T. , Robinson, P. W. , Fowler, M. , & Takahashi, A. (2013). Unravelling the mysteries of a mesopelagic diet: A large apex predator specializes on small prey. Functional Ecology, 27, 710–717. 10.1111/1365-2435.12083

[ece36343-bib-0083] Negus, V. (1958). The comparative anatomy of the nose and paranasal sinuses Livingstons. Edinburgh, U.K: E. & S. Livingstone

[ece36343-bib-0084] Nevitt, G. (1999). Olfactory foraging in Antarctic seabirds: A species‐specific attraction to krill odors. Marine Ecology Progress Series, 177, 235–241. 10.3354/meps177235

[ece36343-bib-0085] Nevitt, G. , Reid, K. , & Trathan, P. (2004). Testing olfactory foraging strategies in an Antarctic seabird assemblage. Journal of Experimental Biology, 207, 3537–3544. 10.1242/jeb.01198 15339950

[ece36343-bib-0086] Nevitt, G. A. , Veit, R. R. , & Kareiva, P. (1995). Dimethyl sulphide as a foraging cue for antarctic procellariiform seabirds. Nature, 376, 680–682. 10.1038/376680ao

[ece36343-bib-0087] Nordøy, E. S. , & Blix, A. S. (2008). Movements and dive behaviour of two leopard seals (*Hydrurga leptonyx*) off Queen Maud Land, Antarctica. Polar Biology, 32, 263–270.

[ece36343-bib-0088] Nordøy, E. S. , Folkow, L. , & Blix, A. S. (1995). Distribution and diving behaviour of crabeater seals (*Lobodon carcinophagus*) off Queen Maud Land. Polar Biology, 15, 261–268.

[ece36343-bib-0089] Nyholm, E. S. (1975). Observations on the walrus (*Odobenus rosmarus* L.) in Spitsbergen in 1971–1972. Annales De Zoologici Fennici, 12, 193–196.

[ece36343-bib-0090] Odend’hal, S. , & Poulter, T. C. (1966). Pressure regulation in the middle ear cavity of sea lions: A possible mechanism. Science, 153, 768–769. 10.1126/science.153.3737.768 5940898

[ece36343-bib-0091] Oelschläger, H. A. (1992). Development of the olfactory and terminalis systems in whales and dolphins In Doty R.L. & Müller‐Schwarze D. (Eds.), Chemical signals in vertebrates 6 (pp. 141–147). Boston, MA: Springer.

[ece36343-bib-0092] Oelschläger, H. H. A. , & Buhl, E. H. (1985). Development and rudimentation of the peripheral olfactory system in the harbor porpoise *Phocoena phocoena* (Mammalia: Cetacea). Journal of Morphology, 184, 351–360. 10.1002/jmor.1051840309 29969871

[ece36343-bib-0093] Orme, D. , Freckleton, G. T. , Petzoldt, T. , Fritz, S. , Isaac, N. , & Pearse, W. (2013). Caper: Comparative analyses of phylogenetics and evolution in R. R package version 0.5.2. Retrieved from http://CRAN.R‐project.org/package=caper

[ece36343-bib-0094] Parrish, F. A. , Abernathy, K. , Marshall, G. J. , & Buhleier, B. M. (2002). Hawaiian monk seals (*Monachus schauinslandi*) foraging in deep‐water coral beds. Marine Mammal Science, 18, 244–258. 10.1111/j.1748-7692.2002.tb01031.x

[ece36343-bib-0095] Parrish, F. A. , Craig, M. P. , Ragen, T. J. , Marshall, G. J. , & Buhleier, B. M. (2000). Identifying diurnal foraging habitat of endangered Hawaiian monk seals using a seal‐mounted video camera. Marine Mammal Science, 16, 392–412. 10.1111/j.1748-7692.2000.tb00932.x

[ece36343-bib-0096] Pauly, D. , Trites, A. W. , Capuli, E. , & Christensen, V. (1998). Diet composition and trophic levels of marine mammals. ICES Journal of Marine Science, 55, 467–481.

[ece36343-bib-0097] Peterson, R. S. , & Bartholomew, G. A. (1967). The natural history and behavior of the California sea lion. Special publication (American Society of Mammalogists). Stillwater, OK: American Society of Mammalogists.

[ece36343-bib-0098] Pihlström, H. (2008). Comparative anatomy and physiology of chemical senses in aquatic mammals In Thewissen J. G. M., & Nummela S. (Eds.), Sensory evolution on the threshold: Adaptations in secondarily aquatic vertebrates (pp. 95–109). Berkeley; Los Angeles; London: University of California Press.

[ece36343-bib-0099] Pihlström, H. , Fortelius, M. , Hemilä, S. , Forsman, R. , & Reuter, T. (2005). Scaling of mammalian ethmoid bones can predict olfactory organ size and performance. Proceedings of the Royal Society B: Biological Sciences, 272, 957–962. 10.1098/rspb.2004.2993 PMC1564090 16024352

[ece36343-bib-0100] Ponganis, P. J. (2011). Diving mammals In Terjung R. (Ed.) Comprehensive physiology (pp. 105–121). Hoboken, NJ: John Wiley & Sons, Inc. 10.1002/cphy.c091003 23737181

[ece36343-bib-0101] Ralls, K. , Hatfield, B. B. , & Siniff, D. B. (1995). Foraging patterns of California sea otters as indicated by telemetry. Canadian Journal of Zoology, 73, 523–531. 10.1139/z95-060

[ece36343-bib-0102] Reidenberg, J. S. (2007). Anatomical adaptations of aquatic mammals. The Anatomical Record: Advances in Integrative Anatomy and Evolutionary Biology, 290, 507–513. 10.1002/ar.20541 17516440

[ece36343-bib-0103] Riedman, M. L. , & Estes, J. (1990). The sea otter (*Enhydra lutris*): Behavior, ecology, and natural history. Biological Report, 90, 1–136.

[ece36343-bib-0104] Robinson, P. W. , Costa, D. P. , Crocker, D. E. , Gallo‐Reynoso, J. P. , Champagne, C. D. , Fowler, M. A. , … Yoda, K. (2012). Foraging behavior and success of a mesopelagic predator in the northeast Pacific Ocean: Insights from a data‐rich species, the northern elephant seal. PLoS One, 7, e36728 10.1371/journal.pone.0036728 22615801 PMC3352920

[ece36343-bib-0105] Ross, G. J. B. (1970). Nuzzling behaviour in captive Cape fur seals, *Arctocephalus pusillus* . International Zoo Yearbook, 12, 183–184.

[ece36343-bib-0106] Rowe, T. B. , Eiting, T. P. , Macrini, T. E. , & Ketcham, R. A. (2005). Organization of the olfactory and respiratory skeleton in the nose of the gray short‐tailed opossum *Monodelphis domestica* . Journal of Mammalian Evolution, 12, 303–336. 10.1007/s10914-005-5731-5

[ece36343-bib-0107] Scholander, P. F. (1940). Experimental investigations on the respiratory function in diving mammals and birds. Hvalråd Skrift (No. 22). I kommisjon hos Jacob Dybwad.

[ece36343-bib-0108] Schreer, J. F. , & Kovacs, K. M. (1997). Allometry of diving capacity in air‐breathing vertebrates. Canadian Journal of Zoology, 75, 339–358.

[ece36343-bib-0109] Schreer, J. F. , & Testa, J. W. (1996). Classification of Weddell seal diving behaviour. Marine Mammal Science, 12, 227–250. 10.1111/j.1748-7692.1996.tb00573.x

[ece36343-bib-0110] Shero, M. R. , Goetz, K. T. , Costa, D. P. , & Burns, J. M. (2018). Temporal changes in Weddell seal dive behavior over winter: Are females increasing foraging effort to support gestation? Ecology and Evolution, 23, 11857–11874. 10.1002/ece3.4643 PMC6303723 30598782

[ece36343-bib-0111] Skinner, J. P. , Burkanov, V. N. , & Andrews, R. D. (2012). Influence of environment, morphology, and instrument size on lactating northern fur seal Callorhinus ursinus foraging behavior on the Lovushki Islands, Russia. Marine Ecology Progress Series, 471, 293–308. 10.3354/meps10038

[ece36343-bib-0112] Smith, T. G. (1980). Polar bear predation of ringed and bearded seals in the land‐fast sea ice habitat. Canadian Journal of Zoology, 58, 2201–2209. 10.1139/z80-302

[ece36343-bib-0113] Stenfors, L. E. , Sadé, J. , Hellström, S. , & Anniko, M. (2001). How can the hooded seal dive to a depth of 1000 m without rupturing its tympanic membrane? A morphological and functional study. Acta Oto‐Laryngologica, 121, 689–695. 10.1080/00016480152583629 11678167

[ece36343-bib-0114] Sterling, J. T. , & Ream, R. R. (2004). At‐sea behavior of juvenile male northern fur seals (*Callorhinus ursinus*). Canadian Journal of Zoology, 82, 1621–1637. 10.1139/Z04-136

[ece36343-bib-0115] Stewart, B. S. , & Delong, R. L. (1995). Double migrations of the Northern Elephant Seal, *Mirounga angustirostris* . Journal of Mammalogy, 76, 196–205. 10.2307/1382328

[ece36343-bib-0116] Stewart, B. S. , Petrov, E. A. , Baranov, E. A. , Timonin, A. , & Ivanov, M. (1996). Seasonal movements and dive patterns of juvenile Baikal seals, *Phoca sibirica* . Marine Mammal Science, 12, 528–542. 10.1111/j.1748-7692.1996.tb00065.x

[ece36343-bib-0117] Stirling, I. (1974). Midsummer observations on the behavior of wild polar bears (*Ursus maritimus*). Canadian Journal of Zoology, 52, 1191–1198. 10.1139/z74-157

[ece36343-bib-0118] Stirling, I. (1983). The social evolution of mating systems in pinnipeds. Advances in the Study of Mammalian Behavior, 7, 489–527.

[ece36343-bib-0119] Team RC (2015). R: A language and environment for statistical computing. Vienna, Austria: R Foundation for Statistical Computing.

[ece36343-bib-0120] Thomas, K. , Harvey, J. T. , Goldstein, T. , Barakos, J. , & Gulland, F. (2010). Movement, dive behavior, and survival of California sea lions (*Zalophus californianus*) posttreatment for domoic acid toxicosis. Marine Mammal Science, 26, 36–52. 10.1111/j.1748-7692.2009.00314.x

[ece36343-bib-0121] Thompson, D. , Duck, C. D. , McConnell, B. J. , & Garrett, J. (1998). Foraging behaviour and diet of lactating female southern sea lions (*Otaria flavescens*) in the Falkland Islands. Journal of Zoology, 246, 135–146. 10.1111/j.1469-7998.1998.tb00142.x

[ece36343-bib-0122] Thums, M. , Bradshaw, C. J. A. , Sumner, M. D. , Horsburgh, J. M. , & Hindell, M. A. (2013). Depletion of deep marine food patches forces divers to give up early. The Journal of Animal Ecology, 82, 72–83. 10.1111/j.1365-2656.2012.02021.x 22881702

[ece36343-bib-0123] Tinker, M. T. , Costa, D. P. , Estes, J. A. , & Wieringa, N. (2007). Individual dietary specialization and dive behaviour in the California sea otter: Using archival time‐depth data to detect alternative foraging strategies. Deep Sea Research Part II: Topical Studies in Oceanography, 54, 330–342. 10.1016/j.dsr2.2006.11.012

[ece36343-bib-0124] Upham, N. S. , Esselstyn, J. A. , & Jetz, W. (2019). Inferring the mammal tree: Species‐level sets of phylogenies for questions in ecology, evolution, and conservation. PLOS Biology, 17, 1–44. 10.1371/journal.pbio.3000494 PMC6892540 31800571

[ece36343-bib-0125] Van Valkenburgh, B. (1990). Skeletal and dental predictors of body mass in carnivores In MacFadden B. J., & Damuth J. (Eds.), Body size in mammalian paleobiology: Estimation and biological implications (pp. 181–205). Cambridge, UK: Cambridge University Press.

[ece36343-bib-0126] Van Valkenburgh, B. , Curtis, A. , Samuels, J. X. , Bird, D. , Fulkerson, B. , Meachen‐Samuels, J. , & Slater, G. J. (2011). Aquatic adaptations in the nose of carnivorans: Evidence from the turbinates. Journal of Anatomy, 218, 298–310. 21198587 10.1111/j.1469-7580.2010.01329.x PMC3058216

[ece36343-bib-0127] Villegas‐Amtmann, S. , Jeglinski, J. W. E. , Costa, D. P. , Robinson, P. W. , & Trillmich, F. (2013). Individual foraging strategies reveal niche overlap between endangered Galapagos Pinnipeds. PLoS One, 8, e70748 10.1371/journal.pone.0070748 23967096 PMC3744541

[ece36343-bib-0128] Wall, S. M. , Bradshaw, C. J. A. , Southwell, C. J. , Gales, N. J. , & Hindell, M. A. (2007). Crabeater seal diving behaviour in eastern Antarctica. Marine Ecology Progress Series, 337, 265–277. 10.3354/meps337265

[ece36343-bib-0129] Watanabe, Y. (2006). Body density affects stroke patterns in Baikal seals. The Journal of Experimental Biology, 209, 3269–3280. 10.1242/jeb.02402 16916962

[ece36343-bib-0130] Watanabe, Y. Y. , Baranov, E. A. , & Miyazaki, N. (2015). Drift dives and prolonged surfacing periods in Baikal seals: resting strategies in open waters? The Journal of Experimental Biology, 218, 2793–2798. 10.1242/jeb.125898 26139663

[ece36343-bib-0131] Watanabe, Y. , Baranov, E. A. , Sato, K. , Naito, Y. , & Miyazaki, N. (2004). Foraging tactics of Baikal seals differ between day and night. Marine Ecology Progress Series, 279, 283–289. 10.3354/meps279283

[ece36343-bib-0132] Welsch, U. , Ramdohr, S. , Riedelsheimer, B. , Hebel, R. , Eisert, R. , & Plötz, J. (2001). Microscopic anatomy of the eye of the deep‐diving Antarctic Weddell seal (*Leptonychotes weddellii*). Journal of Morphology, 248, 165–174. 10.1002/jmor.1027 11304747

[ece36343-bib-0133] Werner, R. , & Campagna, C. (1995). Diving behaviour of lactating southern sea lions (Otaria flavescens) in Patagonia. Canadian Journal of Zoology, 73, 1975–1982. 10.1139/z95-232

[ece36343-bib-0134] Wiig, Ø. , Gjertz, I. , Griffiths, D. , & Lydersen, C. (1993). Diving patterns of an Atlantic walrus *Odobenus rosmarus* rosmarus near Svalbard. Polar Biology, 13, 71–72.

[ece36343-bib-0135] Ylönen, H. , Sundell, J. , Tiilikainen, R. , Eccard, J. A. , & Horne, T. (2003). Weasels’ (*Mustela nivalis nivalis*) preference for olfactory cues of the vole (*Clethrionomys glareolus*). Ecology, 84, 1447–1452. 10.1890/0012-9658(2003)084[1447:WMNNPF]2.0.CO;2

[ece36343-bib-0136] Yurkowski, D. , Semeniuk, C. , Harwood, L. , Rosing‐Asvid, A. , Dietz, R. , Brown, T. , … Ferguson, S. (2016). Influence of sea ice phenology on the movement ecology of ringed seals across their latitudinal range. Marine Ecology Progress Series, 562, 237–250. 10.3354/meps11950

